# Alzheimer’s Disease Leads to Chronic Pathological Changes and Inflammasome Signaling in an Animal Model of Traumatic Brain Injury

**DOI:** 10.3390/cells15181658

**Published:** 2026-09-14

**Authors:** Erika d. l. R. M. Cabrera Ranaldi, Nathan H. Johnson, Andrew P. Sawaya, Jhianyn R. Herrera, Nadine Ahmed Kerr, Helen M. Bramlett, Robert W. Keane, W. Dalton Dietrich, Juan Pablo de Rivero Vaccari

**Affiliations:** 1 The Miami Project to Cure Paralysis, University of Miami Miller School of Medicine, Miami, FL 33136, USA; edc74@miami.edu (E.d.l.R.M.C.R.); jhianynherrera@gmail.com (J.R.H.); n.kerr@med.miami.edu (N.A.K.); hbramlett@med.miami.edu (H.M.B.); rkeane@miami.edu (R.W.K.); 2 Department of Neurological Surgery, University of Miami Miller School of Medicine, Miami, FL 33136, USA; 3 Department of Cellular Physiology and Molecular Biophysics, University of Miami Miller School of Medicine, Miami, FL 33136, USA; 4 Dr. Phillip Frost Department of Dermatology and Cutaneous Surgery, University of Miami Miller School of Medicine, Miami, FL 33136, USA; asawaya@med.miami.edu; 5 Bruce W. Carter Department of Veterans Affairs Medical Center, Miami, FL 33125, USA

**Keywords:** inflammasome, Alzheimer’s disease, ASC, traumatic brain injury, caspase-1, sex differences

## Abstract

Traumatic brain injury (TBI) is a significant public health concern, often resulting in long-term impairments. TBI is also a known risk factor for Alzheimer’s disease (AD), with both conditions sharing features such as inflammasome dysregulation. Inflammasome proteins are carried in extracellular vesicles (EVs) derived from blood plasma and serum. Our previous work showed that TBI in the context of AD acutely increases inflammasome activation and accelerates pathology. However, chronic effects of TBI in AD, particularly regarding sex differences, remain poorly characterized. Here, we used 3xTg-AD mice and wild-type (WT) controls, which underwent moderate controlled cortical impact (CCI) or sham surgery at 5 months of age. Mice were sacrificed at 3 months post-injury for biochemical and histopathological analysis. Cortical and hippocampal lysates were analyzed for inflammasome proteins and inflammatory cytokines via immunoblotting, while pathological markers were assessed using Ella Simple Plex technology and histopathology. In a separate cohort, 3xTg mice received moderate CCI and were sacrificed 7 days post-injury to evaluate sex differences. Cortical lysates were tested for pro-inflammatory cytokines and pathological markers via electrochemiluminescent immunoassay, and EVs were analyzed using mass spectrometry. AD-TBI mice showed increased expression of inflammasome proteins, pro-inflammatory cytokines, and glial fibrillary acidic protein (GFAP). TBI mice displayed elevated neurofilament light in the cortex, with both GFAP and neurofilament light co-localizing with inflammasome proteins in the perilesional cortex. Moreover, AD-TBI mice exhibited reduced cortical and hippocampal volumes compared to WT-TBI mice. In the sex differences cohort, EVs from male and female AD-TBI mice showed distinct patterns in upstream regulators and signaling pathways. Female-derived EVs showed greater activation of AD-related pathways. Sex-specific differences were also observed in cortical pro-inflammatory cytokine expression, although GFAP and amyloid-β levels were not significantly different between sexes. This study demonstrates that AD leads to changes in chronic inflammasome expression and neurodegenerative pathology, with marked sex-dependent differences in inflammatory and signaling responses in AD with TBI. These findings underscore the importance of considering both AD comorbidity and biological sex in the evaluation of TBI outcomes and therapeutic strategies.

## 1. Introduction

Traumatic brain injury (TBI) affects an estimated 69 million individuals worldwide [1] and leads to nearly 70,000 deaths each year in the United States [2]. Similarly, Alzheimer’s disease (AD) also leads to significant lifetime burden on individuals and is an ever-rising issue in society, with global cases estimated to reach 152 million by 2050 [3]. In TBI, the primary injury is the result of acute impact to the brain and is characterized by disruption in neurovascular networks, diffuse white matter injury, neuronal cell death, and the release of pro-inflammatory factors [4]. Secondary injury incites chronic dysfunction [5], a primary facet being inflammatory dysregulation. The release of pro-inflammatory factors and of damage-associated molecular patterns (DAMPs) or pathogen-associated molecular patterns (PAMPs) induces the activation of the innate immune system [6,7]. The primary hallmarks of AD are phosphorylated tau neurofibrillary tangles and amyloid-β (Aβ) plaques. Tau is a neuronal cytoskeletal protein that becomes hyperphosphorylated in AD, leading to cellular damage, homeostatic disruption, and inflammation [8]. Insoluble Aβ can induce glial activation and inflammatory processes when it aggregates into plaques [9]. Moreover, TBI is an established risk factor for AD development [10]. Furthermore, TBI patients demonstrate elevated levels of phosphorylated tau in plasma [11] and have increased Aβ plaque burden after injury [12,13,14].

AD and TBI exhibit chronic inflammatory responses, in part through the innate immune system. Inflammasomes are part of innate immune responses that are elevated in TBI and AD. The inflammasome is composed of a sensor protein such as nucleotide oligomerization domain (NOD)-like receptors (NLRs), which are responsible for sensing DAMPs, the adaptor protein apoptosis-associated speck-like protein containing a caspase recruitment domain (ASC), and the effector caspase-1 [15]. Upon activation by DAMPs, the inflammasome oligomerizes, leading to auto-proteolytic cleavage of caspase-1. Caspase-1 cleaves the pro-inflammatory cytokines interleukin(IL)-1β and IL-18 into their active forms as well as cleaving Gasdermin-D (GSDMD), leading to pyroptotic cell death and the release of IL-1β and IL-18 [16,17]. Other alternative inflammasome pathways can lead to pyroptotic cell death, referred to as non-canonical inflammasomes. Non-canonical inflammasomes follow a different pattern of activation, as they are directly activated by DAMP/PAMP interaction with caspases [18,19]. Non-canonical-associated caspases include caspase-8 and caspases-4/5/11 (4/5 correspond in humans and 11 in rodents). Caspases-4/5/11 are activated by detection of bacterial lipopolysaccharide (LPS), with their signaling leading to direct processing of GSDMD and associated pro-inflammatory cytokines [19]. Meanwhile, caspase-8 is also involved in apoptotic cell death pathways, but it can also regulate inflammasome-led pyroptotic cell death [18]. Non-canonical inflammasome activation can also regulate and induce classical inflammasome recruitment and signaling [20]. Inflammasomes are activated in the cerebral cortex of rodent models of both AD and TBI [21,22]. Furthermore, TBI patients demonstrate increased serum concentrations of ASC and caspase-1 acutely after injury, which are associated with worsened outcomes up to 6 months after TBI [23,24]. In AD, serum ASC and IL-18 levels are significantly elevated in patients, with ASC exhibiting a reliable sensitivity as a biomarker [25].

Inflammasome components can also be transported as cargo through extracellular vesicles (EVs), membrane-enveloped bioparticles released from all cells in the mammalian system. Inflammasome-containing EVs have been shown to be released after TBI from the central nervous system (CNS) [26]. EVs have been detected in the peripheral circulation after TBI and can contribute to systemic dysfunction after injury [27,28,29]. TBI-induced plasma-derived EVs are known to contribute to increased blood–brain barrier breakdown, neutrophil infiltration, and lesion volume in a mouse model of TBI [30]. AD mouse models also show increased levels of inflammasome-containing EVs [27]. EVs themselves are involved in the clearance of Aβ in healthy conditions; however, EVs released from CNS cells can aid in the accumulation and propagation of Aβ plaques [31].

It is well accepted that several sex discrepancies exist in TBI outcomes, with women faring worse than men in mild-to-moderate TBI but better in moderate-to-severe cases [32]. A cross-sectional study found that women who had endured a TBI presented greater plasma tau levels, as well as a greater severity of post-concussive symptoms and a lower health-related quality of life [33].

Pre-clinical work has shown worse performance in cognitive tasks in male mice and greater lesion size after TBI; however, there is still great variance depending on the age and injury model used [34]. As for inflammatory markers, female rats that underwent TBI showed increased astrogliosis and microgliosis post-injury, with changes in cellular signaling underlying observed variations [35]. There is also evidence that progesterone can act as a neuroprotective agent after TBI [36,37]. Fluctuations in estrogen and its consequent decline after menopause have been theorized to contribute to AD development [38,39,40], which is supported by the exacerbation of AD pathology in mouse models that have undergone ovariectomy [41,42,43]. Furthermore, aged women, who have lower levels of endogenous ovarian hormones, are also at a higher risk for adverse outcomes after TBI [44]. In addition, females are twice as likely to develop AD [45,46] and present neurofibrillary tangle pathology associated with clinical severity in post-mortem studies [47,48,49]. Furthermore, women with mild cognitive impairment also experience accelerated progression towards AD compared to men [50]. Therefore, it is integral to understand sex differences that may affect pathology.

We have previously shown that in a murine model of AD-TBI, AD worsens post-TBI inflammasome activation and microglial reactivity up to a week post-injury [21]. Here, we sought to extend the results and examine how other relevant variables are altered in AD-TBI. Specifically, we were interested in examining sex differences in AD-TBI. As the aforementioned study highlighted the effect of AD-TBI on inflammasome signaling up to 7-weeks post-injury, in this study, we selected this time point to assess changes in inflammatory signaling between sexes in the AD-TBI model. Alongside sex differences, we were also interested in examining a more long-term time point compared to prior studies, which showed that TBI can lead to increased white matter injury that persists 3 months after TBI in mice and leads to sustained cognitive deficits at that timepoint [21]. In the R1.40 mouse model of AD, it was shown that TBI also leads to increased lesion volume at 120 days post-injury when compared to non-AD mice, as well as noticeable microglial and macrophage activation [51]. Similarly, we have previously detected variations in cognitive behavior at 3 months in AD-TBI [21] but did not assess the overall inflammatory phenotype of these mice nor assess pathological markers at this timepoint. Therefore, to expand on our previous findings, we selected a 3-month time point to probe for changes in AD pathological markers and inflammasome activation.

Thus, the focus of the current study is to determine the chronic pathological and inflammatory profile of TBI as a disease modifier for progressive AD changes in a transgenic mouse model consistent with TBI being a risk factor for AD and to characterize TBI with AD by examining sex differences. Therefore, a cohort of 5-month-old 3xTg and WT mice underwent TBI, and 3 months post-injury, inflammasome activation and cerebral pathology were assessed.

## 2. Materials and Methods

### 2.1. AD Animal Model

The 3xTg (B6;129-Tg(APPSwe,tauP301L)1Lfa *Psen1^tm1Mpm^*); The Jackson Laboratory, Bar Harbor, ME, USA) mouse strain was used alongside their accompanying B6129SF2 wild-type (WT) controls to model AD. The 3xTg mouse model harbors 3 human transgenic mutations that have been established in AD: *APP*-Swedish, *PSEN1* M146MV, and *MAPT* P301L. Animals demonstrate intracellular Aβ plaque deposition in the cortex at 3–4 months of age and in the hippocampus at 6 months [52,53]. Extracellular deposition of Aβ is first detected in the frontal cortex at 6 months, with astrocytes co-localizing with the deposits [53]. Additionally, 3xTg animals exhibit tau expression at 12 months in the hippocampus, followed by cortical expression and aggregate formation. 3xTg mice present with cognitive impairment similar to the AD presentation, starting with retention deficits that are followed by long-term and short-term memory impairments at 4 and 6 months, respectively [52]. All mice were 5 months of age at the time of surgery, weighing 20 to 40 g, and were split evenly between males and females. Animals were randomly assigned to experimental groups using stratified random sampling, and power analysis was completed to select an appropriate sample size.

### 2.2. Controlled Cortical Injury (CCI)

Animals were first weighed and then anesthetized by administration of ketamine (100 mg/kg) and xylazine (10 mg/kg) via intraperitoneal injections for anesthetic purposes as previously described [21,54,55,56] and according to IACUC protocol #IPROTO202100000158. Petroleum jelly was used to protect the eyes during surgery. Animals were fixed on a stereotaxic frame with ear bars and a bite plate to keep the head immobile during procedures. An incision on the head was made with surgical scissors to expose the skull. Then, a craniotomy was completed using a surgical drill to remove a 4 mm section of the skull 0.5 mm away from midline, between lambda and bregma, to a depth of the dura mater. The CCI device was calibrated and tested before firing; afterwards, the animal was placed under the machine, with the piston aligned to impact the exposed brain area. A moderate CCI was performed with a speed of 4 m/s for a 150 ms duration at a depth of 0.8 mm [57,58,59]. After injury, blood was wiped away with a sterile cotton applicator coated in 75% alcohol, and the scalp was closed with surgical staples. Animals received buprenorphine (0.1 mg/kg) subcutaneously for pain and were placed on a heating pad for recovery. Sham animals underwent craniotomy and drug procedures without CCI. For euthanasia, mice were subjected to 4 to 5% isoflurane followed by decapitation. For the sex differences part of the study, in order to control for changes in hormonal concentration, all female mice were injured during the diestrus stage of the estrus cycle as established by vaginal cytology as described in [60,61].

### 2.3. Tissue Collection

For the chronic TBI study, animals were anesthetized at 3 months post-TBI or sham surgery with 4% isoflurane or with intraperitoneal injections (IP) composed of ketamine and xylazine [62]. For the sex differences cohort, animals were sacrificed at 7 days post-TBI. For immunoblotting, the ipsilateral (right) cerebral cortex and hippocampus were dissected and snap-frozen in liquid nitrogen for storage at −80 °C. The dissected tissue was then homogenized with RIPA buffer with a protease inhibitor cocktail (Sigma-Aldrich, St. Louis, MO, USA) and centrifuged to remove cellular debris. Aliquots of the cortical and hippocampal lysates were then mixed with Laemmli buffer (Bio-Rad, Hercules, CA, USA) and stored in −80 °C until immunoblotting as described in [63]. For the Simple Plex Assay (Biotechne, San Jose, CA, USA), the cortical lysates were prepared the same, but the addition of Laemmli buffer was omitted. For histology and immunohistochemistry, mice were perfused transcardially as previously described [21]. Briefly, animals were perfused with 4% paraformaldehyde (PFA); the brain was then dissected and post-fixed in 4% PFA overnight at 4 °C. Then, brains were placed in 20% sucrose solution until the tissue sank to the bottom prior to sectioning. Sectioning was done using a Leica freezing microtome (Leica Biosystems, Deer Park, IL, USA), with section thickness set to 40 μM.

### 2.4. Mass Spectrometry

To explore whether sex differences exist in immunological signaling pathways that can contribute to differential outcomes in TBI with AD, isolated EVs were processed using liquid chromatography/mass spectrometry as conducted by Tymora Analytical (West Lafayette, IN, USA) [64], using their proprietary EVtrap (EV Total Recovery and Purification) technology that relies on chemical affinity-based methodologies. Briefly, whole blood was collected in ethylenediaminetetraacetic acid (EDTA)-coated tubes and processed immediately for plasma isolation via a 10 min centrifugation at 1800× *g* at 4 °C. Afterwards, 300 uL of plasma was aliquoted and immediately frozen at −80 °C. Isolated plasma samples were shipped in dry ice overnight to Tymora Analytical for processing. EV isolation methods were conducted by Tymora Analytic per its published EVtrap methodology [65,66]. Briefly, plasma was incubated with loading buffers followed by EVtrap beads for 30 min. EVs were then isolated using a magnetic separation rack, beads were washed with 1X PBS, and EVs were eluted with a 10 min incubation with triethylamine. EVs were vacuum-dried for downstream processing. EV protein digestion and phosphopeptide enrichment were completed as previously described [66]. Peptide amount was quantified using Pierce colorimetric peptide analysis to be within 3 μg and 4.1 μg per sample. An equal amount of peptide was loaded for analysis. Exploratory phosphopeptide and protein analyses were completed using an Easy-nLC 1000 (Thermo Fisher Scientific, Waltham, MA, USA) and LTQ-Orbitrap Velos Pro mass spectrometer (Thermo Fisher Scientific) using established processing and parameters [67]. False discovery rate (FDR) was set to 1%.

### 2.5. Electrochemiluminescent Immunoassay (ECLIA)

To quantify cerebral concentration of pro-inflammatory cytokines and neuropathological proteins, undiluted cerebral cortical lysates were probed using ECLIA. Neuroinflammatory protein concentrations were analyzed using the V-PLEX Neurology Panel 1 Kit; the V-PLEX Pro-Inflammatory Panel 1 Kit was used for quantification of pro-inflammatory cytokines, and the V-PLEX Aβ Peptide Panel (Meso Scale Discovery, Rockville, MD, USA) was used for AD neuropathology as previously described [21].

### 2.6. Simple Plex Assay

The Ella System (Biotechne) was used to determine the concentration of neurofilament-light (NfL) and glial fibrillary astrocyte protein (GFAP) from cortical lysates of AD and WT mice with or without TBI. For assay preparation, 35 μL of cortical lysates were diluted in 35 μL of diluent reagent. Subsequently, 50 μL of the diluted sample was added to the wells of a CART, while 1 mL of washing buffer was added to separate wells in the CART as described in [68]. The calculated concentrations from the cortical lysates were obtained through the Runner Software (version 3.9.0.28), with further analytical procedures undertaken through the Simple Plex Explorer program (version 3.9.0.28).

### 2.7. Immunoblotting

Immunoblotting was performed as previously described [63]. Tissue samples were first boiled and then separated on 4–20% Tris-TGX stain-free citron gels (Bio-Rad). Gels were transferred onto polyvinylidene difluoride (PVDF) membranes utilizing Turbo Transfer mixed molecular weight protocol (Bio-Rad). Membranes were blocked in blocking buffer (Bio-Rad). Membranes were incubated in antibodies (1:1000 dilution) against IL-1β (Novus Biologicals, Centennial, CO, USA), NLRP3 (Novus Biologicals), NLRP1 (Novus Biologicals), ASC (Santa Cruz Biotechnology, Dallas, TX, USA), caspase-1 (Novus Biologicals), caspase-11 (R&D Systems, Minneapolis, MN, USA), caspase-8 (Novus Biologicals), and β-actin (Sigma-Aldrich). Membranes were washed in TBS-T prior to exposure to secondary antibodies and imaging reagents. After primary antibody exposure, membranes were incubated in secondary antibodies containing horseradish peroxidase: IgG anti-rabbit (Cell Signaling Technology, Danvers, MA, USA) or IgG anti-mouse (Cell Signaling). Membranes were imaged using a ChemiDoc Touch imaging system (Bio-Rad) with results analyzed utilizing Image-Lab (Bio-Rad) software. The amount of total density of the proteins of interest was determined by measuring specific band intensity and normalizing it to total lane protein density of a stain-free gel prior to transfer or to β-actin protein expression.

### 2.8. Histology

Dissected whole brains were placed in formalin and allowed to fix for 1 week prior to paraffin embedding. Brains were blocked, paraffin-embedded, and then sectioned coronally at 10 um thickness at 500 um intervals as previously described utilizing a microtome (Leica Biosystems) [69]. Sections were mounted on slides and allowed to dry prior to staining. Slides were dewaxed and rehydrated by going through 4 min intervals of 3× xylene, 3× 100% ethanol, 2× 95% ethanol, 1× 75% ethanol, and 1× dH_2_O. Slides were stained for 4 min in hematoxylin and 15 s in eosin and then cleared through the reverse of the rehydrating steps (4 dips per step) and coverslipped. Slides were stored at room temperature until use. Brain volume was determined through tracing of ipsilateral and contralateral cortex and hippocampal sections at 4× magnification using a brightfield (Evident Scientific, Needham, MA, USA) microscope and Neurolucida (MBF Bioscience, Willinston, VT, USA) software (version 7.50.1). Volume was calculated by adding the total area of all contoured sections for each respective brain region, normalized to total whole brain volume.

### 2.9. Statistical Analysis

Data were analyzed with Prism (Version 9.5.0). The robust regression and outlier removal (ROUT) method was used to detect and remove possible extreme outliers; the Q value was set to 0.2% and we found no outliers in any experimental group. Afterwards, normality was determined using the Shapiro–Wilk normality test, and descriptive statistics were gathered. Two-tailed unpaired *t*-tests were completed for two-group comparisons of parametric data, while the Mann–Whitney test was used for non-parametric data. Data from greater than two groups were analyzed using a two-way ANOVA with Šídák’s multiple comparisons test. A *p*-value of 0.05 or below was recognized to be statistically significant. Western blot and pathological analyses were completed using investigators blinded to experimental groups.

Pathway Analyses: Pathway, functional, and upstream regulator analyses were performed using Ingenuity Pathway Analysis (IPA; Qiagen, Redwood City, CA, USA). To identify enriched biological themes, differentially expressed genes (DEGs) were mapped to the Ingenuity Knowledge Base. Significance for canonical pathways and biofunctions was determined using Fisher’s exact test, with an initial *p*-value threshold of *p* < 0.05. Furthermore, IPA calculated a Z-score, which predicts the activation state of a pathway or regulator by comparing the observed gene expression changes against a model of expected changes based on the literature. Pathways or regulators with a |Z-score| ≥ 2.0 were considered significantly activated or inhibited.

## 3. Results

### 3.1. EVs from AD-TBI Mice Exhibit Sex Differences in Inflammatory Signaling

**Figure 1:**
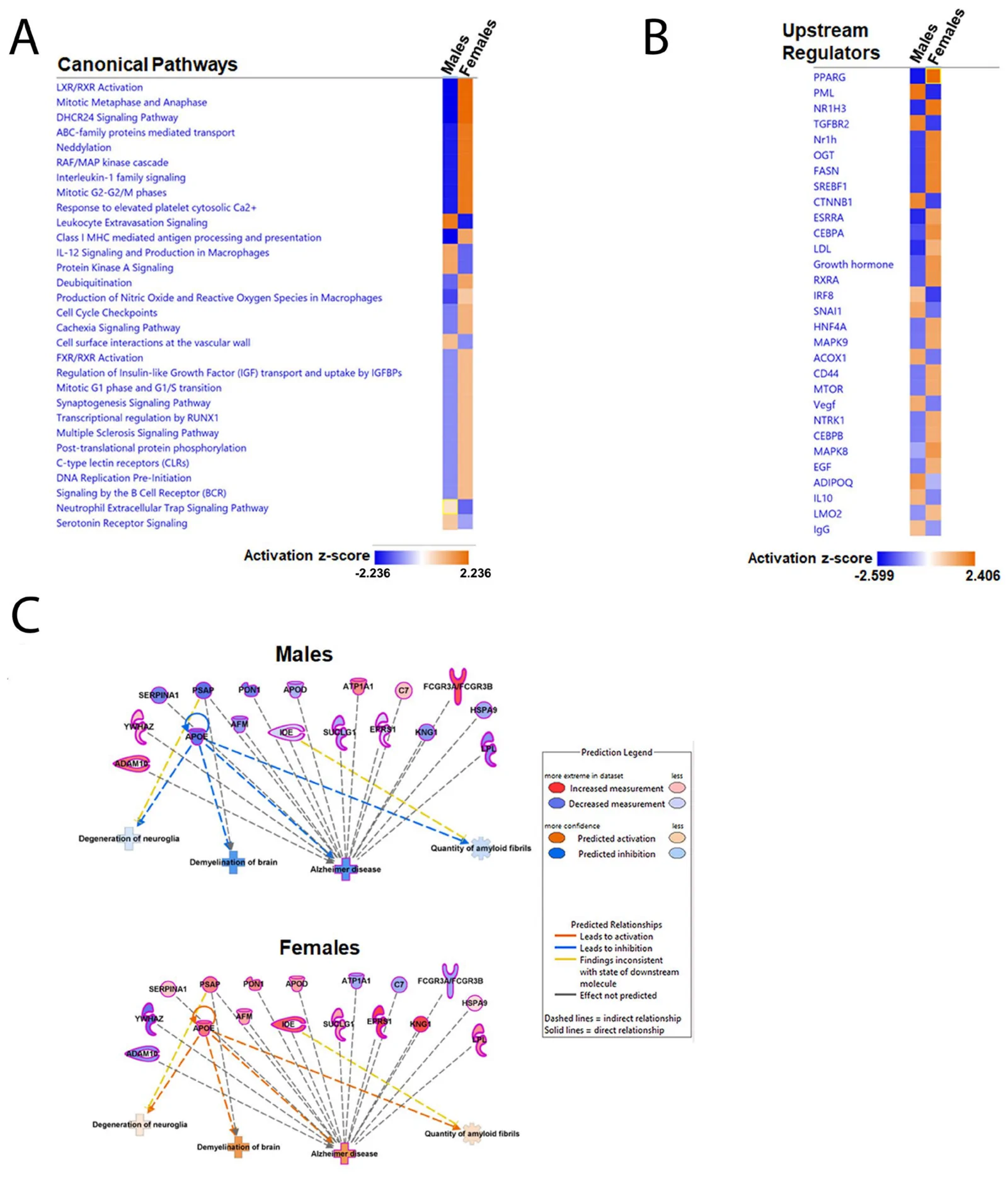
EVs from AD-TBI mice exhibit differential pathway enrichment and inflammatory signaling and AD-associated pathways between sexes. Top canonical enriched pathways (**A**) and upstream regulators (**B**) that differ between AD-TBI male- and female-derived EVs, exhibiting various changes in enrichment between males and females. (**C**) AD-associated pathways in male and female AD-TBI-derived EVs, demonstrating AD-associated proteins and their role in major AD mechanisms. Pathway enrichment analysis was completed with the Ingenuity software package, *p* < 0.05. *N* = 3 per group.

We have previously shown that the inflammatory response in the brain of aged mice differs between males and females [70], and pre-clinical studies have shown that post-injury inflammatory responses differ between sexes [34]. Thus, after determining the effects of AD on acute TBI recovery in previous work, we were interested in examining possible sex differences in TBI with AD at this time point. Furthermore, EVs act as important modulators of inflammatory action through their ability to shuttle cytokines, proteins, and genetic information toward various cell types. Therefore, we conducted exploratory analyses for sex differences in inflammatory signaling using blood plasma-derived EVs processed for mass spectrometry (*n* = 3 per group). Pathway enrichment analysis revealed differences in canonical pathway enrichment between AD-TBI male- and female-derived EVs; females had increased enrichment in liver X receptors (LXR)/retinoid X receptor (RXR) activation, ABC family protein-mediated transport, IL-1 family signaling, neddylation, response to elevated platelet cytosolic calcium, and major histocompatibility complex-I (MHC-I)-mediated antigen processing and presentation (Figure 1A). On the other hand, males had increased enrichment in several processes, including leukocyte extravasation signaling, IL-12 signaling and macrophage production, protein kinase A signaling, and cell surface interactions (Figure 1A). Pathway analysis also examined differences in upstream regulators, with female-derived EVs having greater enrichment in peroxisome proliferator-activated receptor gamma (PPARG), Nuclear Receptor Subfamily 1 Group H Member 3 (NR1H3), and O-GlcNAc transferase (OGT), among others, when compared to male-derived EVs (Figure 1B). Male-derived EVs were upregulated (Figure 1B) in catenin beta 1 (CTNNB1), interferon regulatory factor 8 (IRF8), and snail family transcriptional repressor 1 (SNAI1). Taken together, these indicate that male and female AD-TBI-derived EVs had variable enrichment patterns in regulatory pathways, specifically those involved in immune activity, which has an interesting impact when assessing inflammatory changes between sexes.

### 3.2. AD-Associated Pathways in EVs Differ Between Sexes in TBI with AD

To determine the changes in AD-related signaling pathways, pathway analysis of AD-specific proteins of the isolated EVs was carried out for exploratory studies. Overall, several different proteins associated with AD processes were identified, such as neuroglial degeneration, demyelination, and amyloid fibril formation (Figure 1C). Female-derived EVs exhibited a broad upregulation of AD-associated and demyelination processes, and male-derived EVs had decreases in AD-associated pathways (Figure 1C). Furthermore, male-derived EVs had lower measured apolipoprotein E (APOE), resulting in a predicted inhibition of AD-associated pathways, while female-derived EVs had increased APOE measurements that increased activation of the same pathways. These results suggest that EV signaling is involved in AD-related mechanisms in AD-TBI male and female mice.

### 3.3. No Sex-Dependent Changes Identified in Aβ Species Expression in the Cerebral Cortex of AD Animals 7 Days Post-TBI

To determine whether there were sex differences in Aβ species in the cerebral cortex, cerebrocortical lysates collected at 7 days post-TBI were analyzed by ECLIA for Aβ-38, Aβ-40, and Aβ-42. No differences between male AD-TBI and female AD-TBI mice were found for any of the aforementioned Aβ species (Figure S1A–C), nor in the Aβ-42/Aβ-40 ratio (Figure S1D). Thus, at the assessed time point of 7 days post-TBI, no sex differences were identified in Aβ species expression in the cerebral cortex of AD-TBI mice. Moreover, we also probed for changes in GFAP expression (Figure S1E), but we did not find sex differences at the 7 days post-injury time point.

### 3.4. Female AD-TBI Present Decreased Expression in TNF-α and KC/GRO in the Cerebral Cortex 7 Days Post TBI

**Figure 2:**
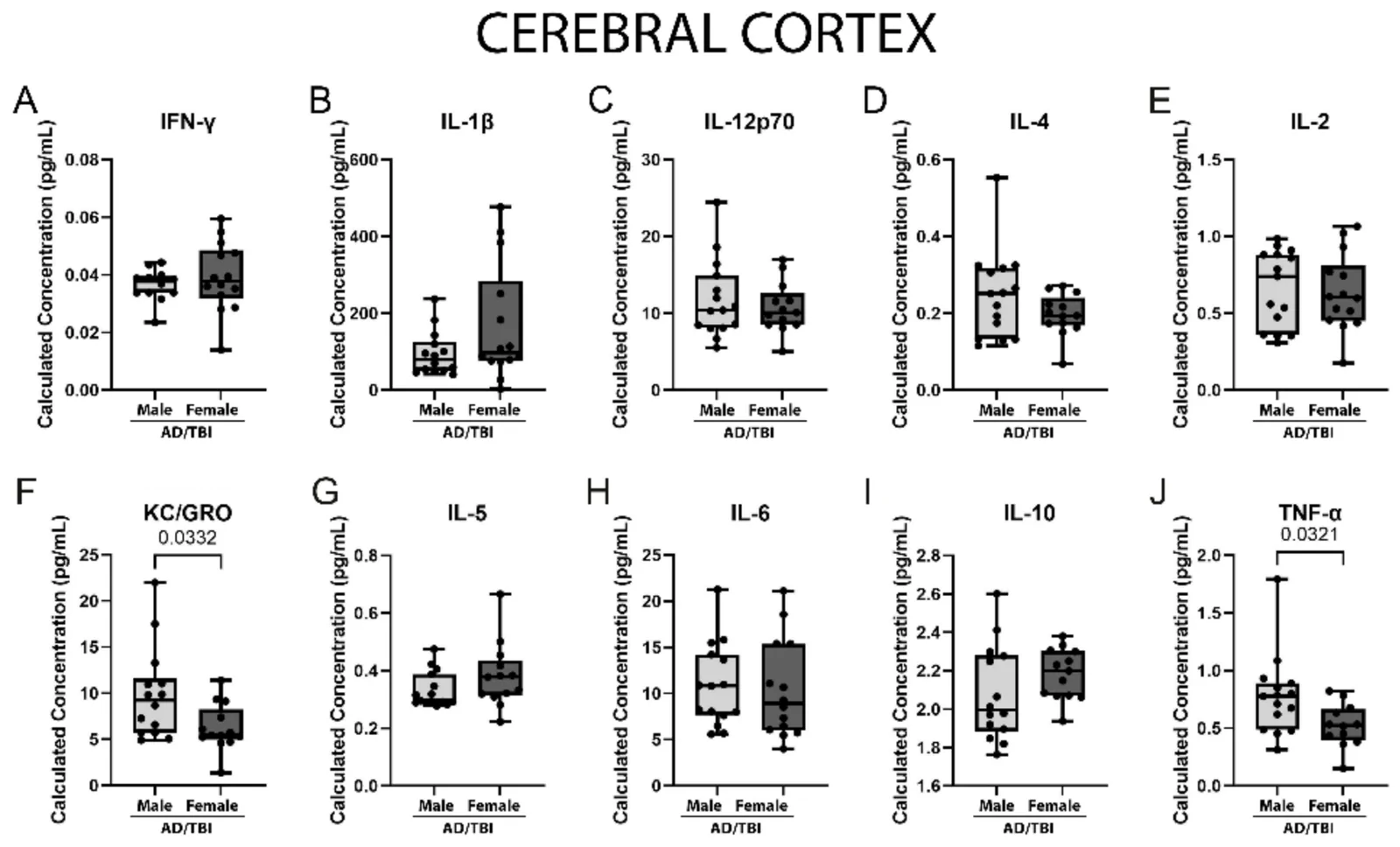
Female AD-TBI mice have reduced expression of pro-inflammatory cytokines KC/GRO and TNF-α in the cerebral cortex 7 days post-TBI. Calculated concentration of several inflammatory cytokines in cerebral cortical lysates 7 days post-injury, normalized to total protein concentration. There was a marked reduction in KC/GRO (**F**) and TNF-α (**J**) in the cerebral cortex of female AD-TBI mice 7 days post-injury when compared to male AD-TBI mice. There were no differences in interferon-γ (IFN-γ; (**A**)), IL-1β (**B**), IL-12p70 (**C**), IL-4 (**D**), IL-2 (**E**), IL-5 (**G**), IL-6 (**H**), or IL-10 (**I**). Data shown as mean ± SEM. Data were analyzed using a Mann–Whitney test between groups. *N* = 14 per group.

Since females tend to present higher levels of inflammation in the aging brain than males [70] and females are more likely to develop AD [71], we also aimed to assess the expression of a panel of pro-inflammatory cytokines in the cerebral cortex of AD mice after TBI (Figure 2). For several cytokines, there was no observed difference in cortical expression at this time point. However, there was a significant difference in keratinocyte chemoattractant/growth-related oncogene (KC/GRO) and tumor necrosis factor-α (TNF-α). Specifically, female AD-TBI mice had a marked reduction in both KC/GRO (Figure 2E) and TNF-α (Figure 2I) expression in the cerebral cortex 7 days post-TBI when compared to male AD-TBI mice. Thus, male and female AD-TBI mice have differing cytokine expression patterns after TBI only in a few key inflammatory cytokines.

### 3.5. TBI Worsens Cortical and Hippocampal Volume Loss 3 Months Post-TBI in AD Mice

**Figure 3:**
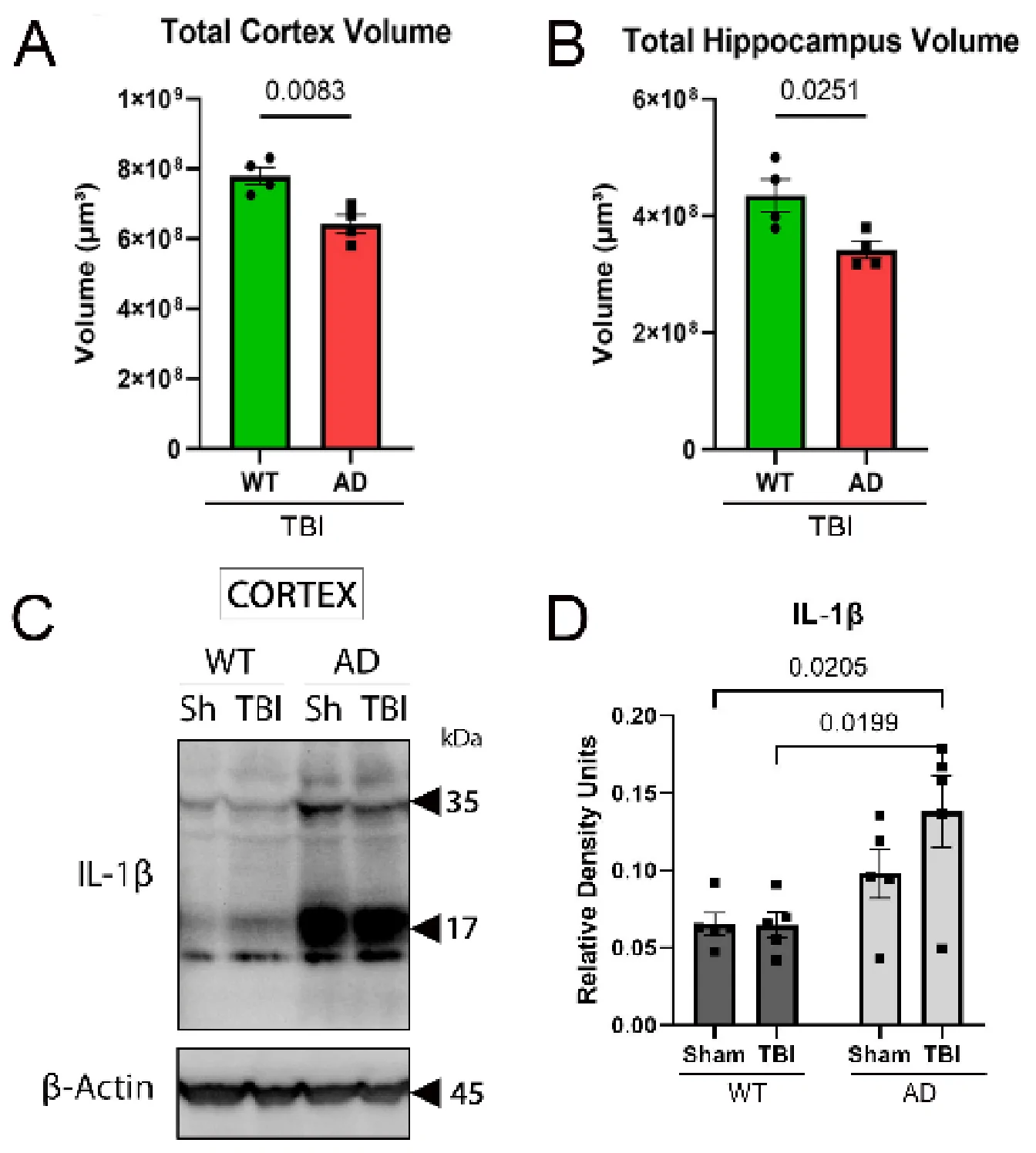
TBI leads to brain volume loss and chronic IL-1β elevation 3 months post-injury in AD mice. (**A**) There was a significant loss in total cortex volume in AD-TBI animals when compared to WT-TBI (*n* = 4); normalized to total brain volume. (**B**) Total hippocampal volume was significantly reduced in AD-TBI mice compared to WT-TBI (*n* = 4); normalized to total brain volume. (**C**) Representative immunoblots normalized to β-actin for IL-1β protein expression in cerebral cortical lysates of AD and WT mice with (TBI) or without (sham) moderate CCI. (**D**) Genetically predisposed AD mice demonstrate significantly higher levels of cleaved IL-1β (17 kDa) post-injury compared to injured WT mice (*n* = 5). Data shown as mean ± SEM. A two-way ANOVA with Šidák’s multiple comparisons test was used to compare groups in Figure (**D**). For (**A**,**B**), an unpaired two-tailed Student’s *t*-test was used.

TBI leads to significant brain atrophy, which is seen in both clinical [72,73] and pre-clinical studies [74,75]. Thus, to determine volumetric changes at 3 months post-TBI in the cortex (Figure 3A) and hippocampus (Figure 3B) of WT and AD mice, whole brains (*n* = 4 per group) were dissected 3 months after moderate CCI (8-month-old AD and WT at time of sacrifice). Dissected brains were then sectioned and stained for hematoxylin and eosin, after which volumetric analyses were conducted to determine cortical and hippocampal volume changes. Volumetric analysis revealed that AD-TBI mice had significantly less cerebral cortical volume (Figure 3A) in comparison to WT-TBI animals at 3 months post-injury. Similarly, AD-TBI animals also demonstrated less hippocampal volume than WT-TBI mice (Figure 3B). Therefore, TBI increases the severity of cortical and hippocampal atrophy in AD mice 3 months after trauma.

### 3.6. IL-1β Is Elevated in the Cerebral Cortex of AD Mice Regardless of Injury Status

We have previously established that the inflammatory cytokine IL-1β is elevated at acute time points in AD mice after TBI [21]. Thus, to determine the levels of IL-1β at a more chronic stage, we dissected the cerebral cortex of mice (*n* = 5 per group) 3 months after sham surgery or moderate TBI. The ipsilateral cortex was homogenized, and cortical lysates were immunoblotted for IL-1β protein expression (Figure 3C). Densitometric analyses revealed a significant increase in IL-1β protein expression in the cortex of AD mice who had undergone TBI compared to the WT-TBI cohort (Figure 3D). There were also increased levels of IL-1β protein in the AD-TBI cohort versus WT-sham mice. However, there were no differences in IL-1β protein expression between AD groups at the chronic stage. Thus, these findings indicate that the protein expression of the pro-inflammatory cytokine IL-1β remains elevated in injured AD mice above both WT groups, indicating an AD genotype effect at play rather than a synergistic TBI change on inflammatory activity.

### 3.7. Canonical Inflammasome Components Are Increased 3 Months in AD Mice

**Figure 4:**
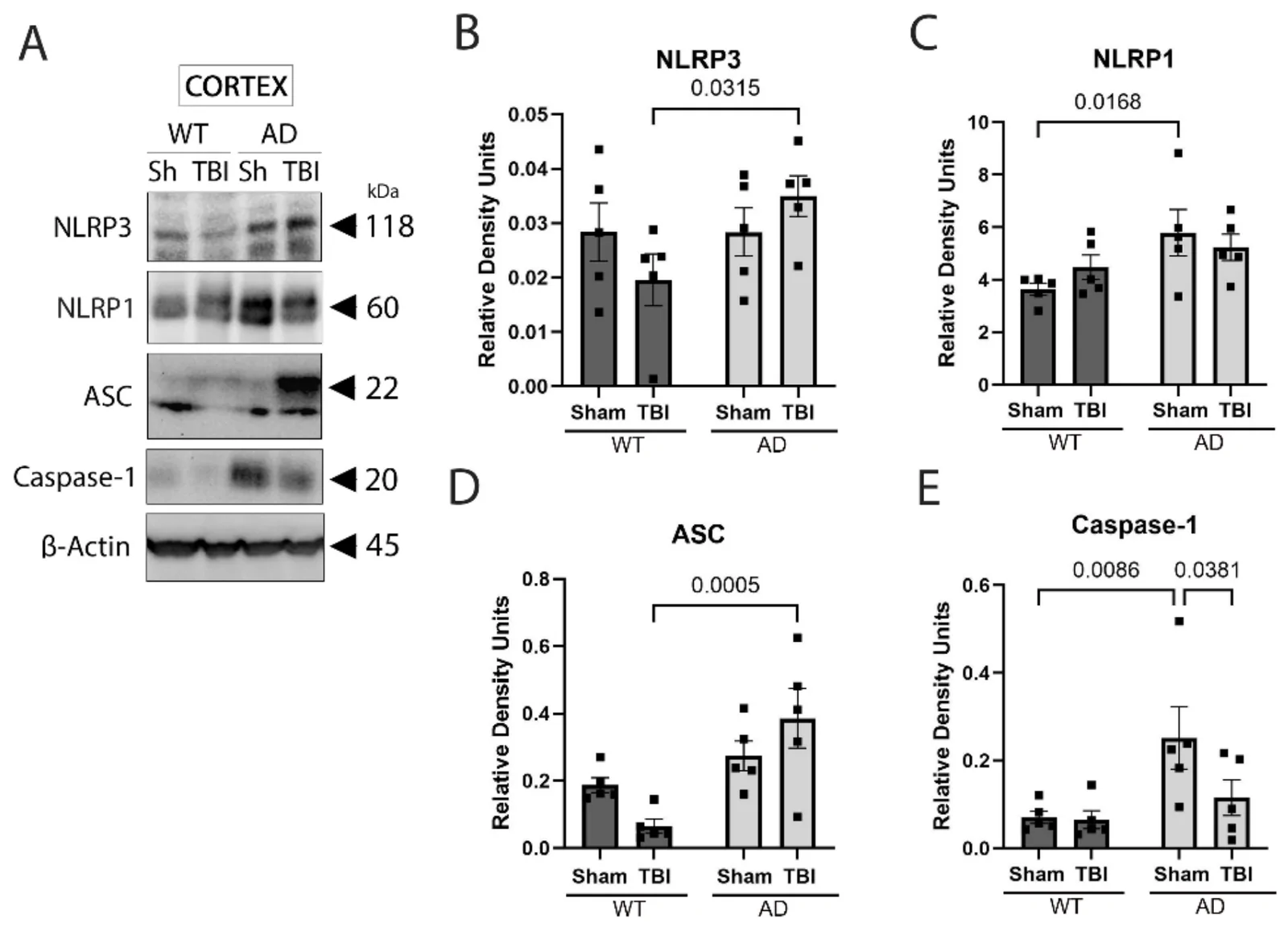
Expression of inflammasome components is increased in genetically predisposed AD mice. (**A**) Representative immunoblots normalized to β-actin for inflammasome signaling protein expression in cerebral cortical lysates of AD and WT mice with or without moderate CCI. (**B**) There was a significant elevation in NLRP3 protein expression in the cortex of AD-TBI versus WT-TBI mice. (**C**) There were no significant changes in NLRP1 levels between the injury groups. (**D**) ASC was significantly higher in the cortex of AD-TBI mice compared to WT-TBI controls. (**E**) There were no significant changes in caspase-1 levels between the injury groups. Data shown as mean ± SEM. A two-way ANOVA with Šidák’s multiple comparisons test was used to compare groups, or a Kruskal–Wallis test with Dunn’s multiple comparisons for non-normally distributed data (caspase-1). *N* = 5 per group with significant *p*-values shown.

Next, we sought to investigate whether inflammasome signaling proteins were increased in AD-TBI mice at chronic time points by immunoblotting lysates of the ipsilateral cerebral cortex (*n* = 5 per group; Figure 4). Immunoblots were performed for the NLRP3 and NLRP1 sensor proteins, the inflammasome adaptor ASC, and the effector protein caspase-1. Immunoblot analyses revealed a significant increase in NLRP3 and ASC (Figure 4B,D) protein expression in AD-TBI mice when compared to WT-TBI animals at 3 months post-injury, indicating greater chronic expression of inflammasome components after TBI in AD mice due to the presence of the AD genotype in general. For NLRP3 and ASC, there was no change in protein expression between AD-TBI and AD-sham groups. In addition, we did not find any significant patterns in NLRP1 (Figure 4C) nor in cleaved caspase-1 (Figure 4E) between the injury groups, indicating a possible alternative pathway for inflammasome signaling at 3 months post-TBI. However, there were differences in caspase-1 expression between AD groups, specifically showing a reduction in caspase-1 expression in AD-TBI in comparison to AD alone, indicating a suppression of canonical inflammasome activity in response to injury. In the case of NLRP1, AD-sham mice had greater protein expression in comparison to WT-sham, but no other differences were detected. Overall, there is an AD-dependent effect on increased canonical inflammasome components, but no injury effect was detected.

To further describe patterns of inflammasome activity in AD-TBI mice and to profile injury severity, immunoblots were also conducted on ipsilateral hippocampal lysates of injured and sham surgery animals of both phenotypes. According to densitometric data, no significant patterns emerged in IL-1β, ASC, or caspase-8 protein expression within the hippocampus between phenotypes after injury (Figure S2). Overall, we show that AD leads to increased inflammatory processes led by inflammasome components regardless of injury status.

### 3.8. Caspase-8 Is Elevated 3 Months AD Mice Regardless of Injury Status

**Figure 5:**
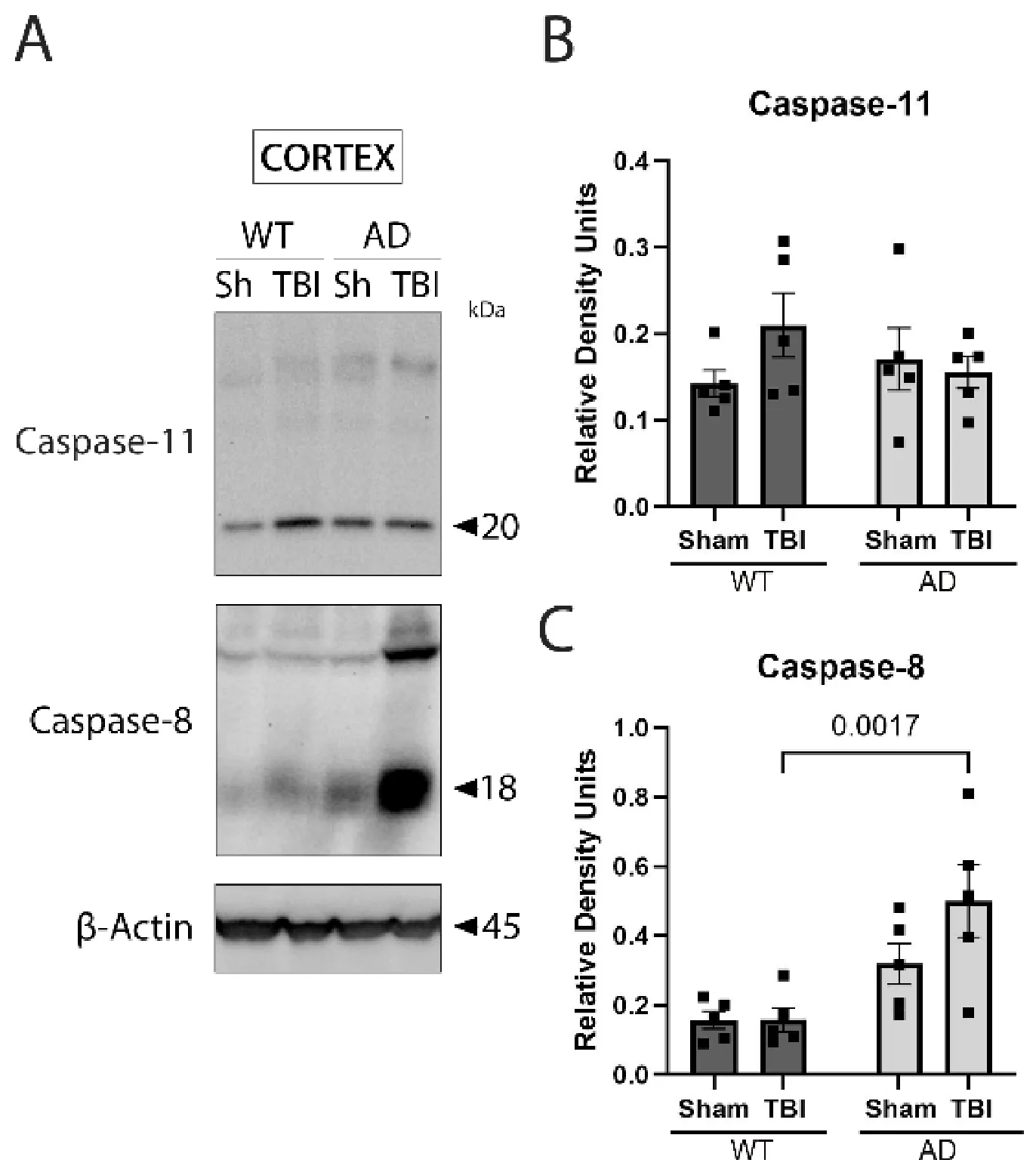
Non-canonical inflammasome expression is significantly increased in the cortex of AD mice. (**A**) Representative immunoblots normalized to β-actin for inflammasome signaling protein expression in cerebral cortical lysates of AD and WT mice with or without moderate CCI. (**B**) There were significantly increased caspase-11 levels in the cortex of AD-TBI mice in comparison to WT-TBI mice. (**C**) Caspase-8 expression was significantly greater in AD animals compared to WT after moderate CCI. Data shown as mean ± SEM. A two-way ANOVA with Šidák’s multiple comparisons test was used to compare groups. *N* = 5 per group with significant *p*-values shown.

As we did not identify a significant increase in canonical inflammasome activation (caspase-1) within the injury groups at 3 months after TBI, we were interested in examining whether the non-canonical inflammasome pathway could be involved in inflammasome activity at this chronic stage (Figure 5). Caspase-11 and caspase-8 are both alternative effector proteins that can modulate non-canonical inflammasome pro-inflammatory cytokine release [76], and we have previously determined that caspase-8 activation increases in AD-TBI mice at acute stages post-injury [21]. Immunoblotting of cortical lysates (*n* = 5 per group) for non-canonical inflammasome signaling proteins did not show changes in caspase-11 activation 3 months after TBI (Figure 5B). However, there was a persistent increase in activated caspase-8 in AD-TBI mice in comparison to WT-TBI (Figure 5C). However, this pattern was not seen to differ between AD groups. Thus, AD mice continue to express greater levels of caspase-8 than WT animals, supporting the involvement of the non-canonical inflammasome in the pro-inflammatory response in the presence of AD-associated genetic changes.

### 3.9. Biomarkers of Neurodegeneration in Mice at Chronic Stages After TBI

**Figure 6:**
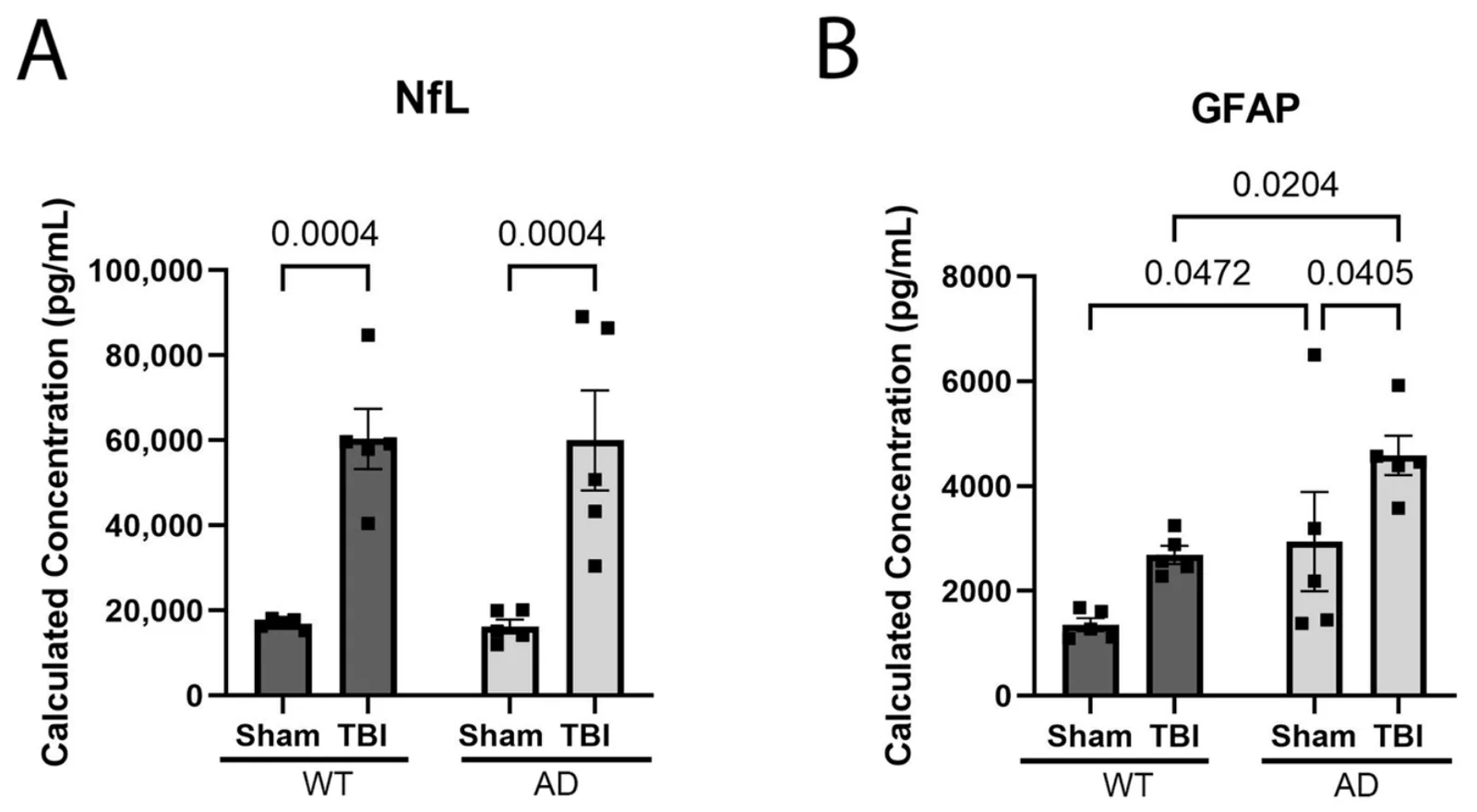
NfL and GFAP are increased in the perilesional cortex 3 months after TBI and demonstrate co-localization with ASC specks. (**A**) Ella assay of cortical lysates from AD and WT mice with or without moderate CCI revealed a significant elevation in calculated concentration (pg/mL) of NfL protein in TBI mice regardless of phenotype. (**B**) Ella assay of cortical lysates indicated a significant increase in the calculated concentration (pg/mL) of GFAP in AD-TBI mice compared to WT-TBI. Data shown as mean ± SEM. A two-way ANOVA with Šidák’s multiple comparisons test was used to compare groups. *N* = 5 per group with significant *p*-values shown.

NfL and GFAP have been investigated as markers for TBI pathology. NfL can indicate neuronal degeneration and axonal destabilization [77], while GFAP expression labels astrocytes, important modulators of the inflammatory response and astrogliosis after TBI [78]. To assess changes in NfL and GFAP, we probed cortical lysates (*n* = 5 per group) of WT and AD mice with or without TBI using a microfluidics assay (Ella, ProteinSimple). The NfL protein levels were significantly elevated in both injury groups compared to their respective sham controls, demonstrating that TBI, regardless of phenotype, leads to increased NfL in the cortex (Figure 6A). For GFAP, sham AD mice had increased levels of GFAP expression when compared to sham WT mice (Figure 6B). Interestingly, GFAP was elevated in AD-TBI when compared to both WT-TBI and AD-sham mice (Figure 6B).

## 4. Discussion

The inflammasome is a key contributor to the inflammatory response after TBI in rodents [79] and humans [80]. TBI and AD have an interlinked relationship: TBI is a risk factor for AD development [81,82] and an accelerator for the onset of AD by 4 years compared to non-TBI AD patients [83]. TBI [84,85] and AD [86,87] have detrimental pro-inflammatory responses that perpetuate cellular damage and pathology, emphasizing the importance of characterizing these inflammatory mechanisms to improve outcomes. Pre-clinically, multiple studies have assessed the intersection of both conditions and their influence on each other’s pathology [25,88]. However, the chronic interactions between TBI and AD predisposition, as well as sex-dependent changes in inflammatory signaling, are less understood.

Here, we studied inflammasome activation by presenting the protein expression of active (cleaved) caspases-1 and -8 as well as IL-1β and found that AD, regardless of injury status, leads to increased inflammasome signaling in the cerebral cortex 3 months post-TBI, as well as to significant changes in cellular pathology. Interestingly, there was differential expression of inflammasome-associated proteins instead of the whole complex, similar to what we have previously detected in the brain of AD donors in the early stages of the disease [89,90,91]. Moreover, a separate cohort of 3xTg mice underwent TBI and were probed for sex differences 7 days post-injury. In this cohort of mice, there were significant changes in EV pathway and AD-associated signaling. Furthermore, we also found that female mice had altered expression patterns of pro-inflammatory cytokines in the cerebral cortex when compared to male mice. Our findings further illustrate a continued, exacerbated inflammatory response and pathological changes seen in TBI with AD, and demonstrate the unique patterns of sex differences within the model.

The activation of the inflammasome in TBI has been well documented. In patients, inflammasome proteins such as ASC and caspase-1 are elevated in the blood after TBI and serve as potential biomarkers, with greater expression of inflammasome components predicting worsened outcomes 6 months after injury [23,80]. In animal studies, inflammasome components are also shown to be elevated in various models of TBI [92,93], and AD is also associated with increased inflammasome protein expression [94]. Furthermore, TBI with AD predisposition can lead to exacerbation of inflammasome activation above AD and TBI controls acutely in the cerebral cortex [21].

We have selected the CCI model of TBI due to its high reproducibility and its ability to induce TBI pathophysiology representative of the human condition. The CCI model uses an impact force that injures a specific area of the cerebral cortex at a pre-selected severity and reliably produces an inflammatory insult, cerebrocortical lesions, axonal injury, and blood–brain barrier damage [95,96] to an extent corresponding to the severity of injury. Animals who undergo CCI also present functional impairments related to memory, learning, and motor control, similar to cognitive changes in the clinical population [57]. However, although the model does have a limitation of requiring surgical intervention to induce a focal lesion directly onto the brain, other models, such as the weight drop model, which does not require incision, have increased mortality and lower reproducibility between injury attempts that are not ideal for our study [95]. In addition, the fluid percussion model shares commonalities with CCI, having a higher reproducibility ratio and also requiring craniotomy for injury [96]; however, that model shares the limitation of creating a more severe injury and increased risk of mortality [97]. Thus, due to these reasons and because we have established AD-TBI interactions in our previous work using the CCI model [21], it was the model of choice for this study.

In terms of sex differences in TBI with AD, we assessed changes in EV-mediated inflammatory signaling. Previous studies have found sex differences in EV signaling in healthy aging, showing lower enrichment in oxidative stress pathways and higher enrichment in fat metabolism in females [98] but also in pathological settings. In the 5xFAD mouse model of AD, female brain-derived EVs have been demonstrated to express greater quantities of the CD9 tetraspanin, and these CD9^+^ EVs were found to be more neurotoxic and viable for carrying Aβ than those of males [99]. In our case, we identified several upstream regulators and canonical pathways in EVs that varied between males and females. Furthermore, female AD-TBI-derived EVs were downregulated in several inflammatory pathways, including those associated with neutrophil and macrophage activity. Additionally, female AD-TBI-derived EVs had increased enrichment in AD-associated pathways, leading to increased activation of AD processes. However, we did not find sex differences in the expression of Aβ species in the cerebral cortex at this time point, though there was reduced pro-inflammatory cytokine expression when compared to male AD-TBI mice. Together, our findings support the hypothesis that TBI with AD leads to an exacerbated pro-inflammatory response and pathological change and demonstrates the at the intersection of both conditions, sex-dependent changes in pro-inflammatory signaling are also at play.

In addition, as a hypothesis-generating study, we carried out a pathway enrichment analysis that illuminated various signaling components. The sample size for our mass spectrometry analysis was selected as the analysis has a high sensitivity and throughput per previous publications, thus allowing us to reach enough statistical power to obtain statistically significant differences [64,100,101,102,103,104]. However, future studies are needed to expand on these exploratory studies to validate the role of the relevant analytes in EVs in order to understand their individual contributions to AD pathology after TBI in males and females. Similarly, the present study was not powered to compare the combined effects of genotype, TBI, and sex using a 3-way ANOVA. Therefore, caution should be taken when interpreting the effects of TBI vs. sham in WT and AD mice on EV cargo in males vs. females. This study was powered to analyze the inflammasome-related responses to TBI in AD mice and separately to determine the effects of AD-TBI on EV cargo in males vs. females. Therefore, future studies with a larger cohort of mice are needed to assess the combined interactions between sex, TBI, and AD genotype.

However, our findings suggest several intriguing lines of inquiry for further future mechanistic exploration. Analysis demonstrated enhanced enrichment processes in female AD TBI-derived EVs for LXR/RXR activation, ABC family protein-mediated transport, IL-1 family signaling, neddylation, response to elevated platelet cytosolic calcium, and MHC-I-mediated antigen processing and presentation. As for AD-TBI male-derived EVs, there was greater enrichment in leukocyte extravasation, IL-12, and protein kinase A signaling pathways. Interestingly, APOE, a lipid transporter protein coded by the *APOε* gene in which the ε4 allele is associated with significantly greater AD risk, is a target of LXR signaling [105,106].

Neddylation pathways were also enriched in female-derived EVs. Neddylation is a type of ubiquitin-like modification that can modulate the activation of NF-ΚB-induced pro-inflammatory cytokine gene expression [107]. Neddylation pathways become altered during AD and brain injury and can affect proper protein function [108,109]. Moreover, MHC-I and the IL-1 family of receptors were also different between male and female EVs. MHC-I is an important mediator of innate immunity activation, and the literature describes its role in AD development and peripheral immune cell infiltration [110]. Similarly, IL-1 pathways, of which IL-1β is a member, are critical modulators of the inflammatory response in TBI [111,112] and AD [113,114]. As for males, leukocyte extravasation signaling pathways were upregulated in plasma-derived EVs. Leukocytes infiltrate the CNS during immune activation, which occurs in both AD [115] and TBI. Modulation of infiltrating leukocytes in the CNS in AD and TBI mouse models can affect cognitive impairment and inflammatory signaling [116,117]. Thus, our results point toward new EV-mediated pathways of interest in male and female AD transgenic mice that warrant further investigation and characterization.

Similarly, there was an increase in AD-associated and demyelination pathways in female AD-TBI mice when compared to male AD-TBI EVs. We also found significant enrichment in APOE-mediated processes in female EVs, which resulted in activation of AD pathways; the inverse pattern was observed in males. APOE is well established to be involved in the pathogenesis of AD, as the *APOε4* variant of the protein’s gene is linked to a substantial increase in risk for late-onset AD [118]. Interestingly, female *APOε4* carriers present with more profound cognitive dysfunction compared to male carriers, even in healthy aging [119], and are associated with greater tau pathology in females [120]. Women with mild cognitive impairment also experience accelerated progression towards dementia and AD compared to men [50]. Furthermore, in TBI, female *APOε4* carriers have worse scores in the Glasgow Coma Scale-Extended 24 h post-injury [121]. Our results indicating higher APOE regulatory mechanisms and AD pathway enrichment in females fall in line with the previous literature and demonstrate that AD-TBI mice continue to express sex discrepancies in AD signaling.

To further understand changes in AD pathology Aβ expression, we also examined total cortical expression of several Aβ species in male and female AD mice 7 days post-TBI. The previous literature has trended towards increased Aβ deposition in female AD patients [49,122]. In our study, we examined the cortical concentration of Aβ-38, Aβ-40, and Aβ-42 species, as well as the Aβ-42/40 ratio, which has been well documented as a diagnostic tool for AD [123]. We did not find any significant differences in any of the Aβ species or in the Aβ-42/40 ratio between male and female mice 7 days post TBI in the cerebral cortex. It is possible that the lack of sex differences in Aβ expression levels seen in this study could be due to the time point assessed, as prominent Aβ species changes between sexes have been seen primarily from 12 months of age and further [124].

There have also been established sex differences in cytokine expression in AD-only and WT-only groups, demonstrating variable expression patterns between sexes in both conditions. For example, a study found that female mice had greater IL-1β expression at 4 h and 7 days post-TBI, though the opposite pattern appeared at 1 and 3 days post-injury [125]. The same results were seen for TNF expression between female and male mice post-TBI. For other cytokines, TGF-β and Arg1 similarly peaked at differing time points between males and females. Another study looking at microglia-specific cytokine signaling also found post-CCI differences between males and females and that time with increased IL-1β expression at 1–7 days post-TBI, a 1–3-day peak in TNF signaling [126], and overall higher microglial and macrophage activation post-injury are seen [127]. Moreover, male mice who underwent TBI had higher levels of TNF at 7 days post-TBI and of TGF-β at 1–3 days post-injury. In the case of AD, 3xTg mice also exhibited sex differences in inflammatory cytokine expression. A longitudinal study found that 3xTg male mice had increased levels of IL-12, IL-10, and C-X-C motif ligand (CXCL) 2 at 18 months of age, while females had increased levels of CXCL9 and CXCL10 at 24 months of age [128]. Thus, we sought to add to the literature by characterizing the intersection of TBI and AD while focusing on differences in inflammatory cytokine expression between sexes in these conditions. We found a sex difference in two of the assessed cytokines, KC/GRO and TNF-α, both of which were increased in the cerebral cortex of male AD mice when compared to female AD mice 7 days post-TBI. KC/GRO, also known as CXCL1, is a pro-inflammatory cytokine with roles in neutrophil trafficking [129]. In TBI, KC/GRO has been demonstrated to be increased in the serum of patients and to be a predictor of poorer outcomes [130]. In other forms of brain injury such as cerebral ischemia, increased KC/GRO has also been identified [131], and ablation of its activity can reduce neutrophil invasion [132]. Sex differences in KC/GRO expression have also been seen in the 3xTG mouse model, with male mice presenting with increased levels of the protein at an earlier time course when compared to female mice [128]. Thus, KC/GRO is an established marker of pro-inflammatory signaling in several forms of brain injury, and our results of decreased expression in female AD-TBI mice showcase an established sex difference, characterizing the inflammatory response in the complexity of TBI with AD predisposition.

As for TNF-α, the literature has well documented increases in protein expression post-TBI, both clinically and pre-clinically [133,134,135], and shown a role of TNF-α as a modulator in post-injury pathology and functional recovery [136]. In animal models of TBI, adult male mice present upregulated expression of TNF-α at 1 and 3 days post-TBI [125]. In AD, TNF-α is also overexpressed in 3xTg mouse models from 12 months on in the hypothalamus [137]. In our study, we continued the established trend of increased pro-inflammatory cytokine expression in the cerebral cortex of male mice 7 days post-injury, extending our understanding to TBI with AD. It is interesting to note that all female mice were injured during diestrus, a period of the estrous cycle where progesterone levels are elevated [138]. Progesterone has been shown to be neuroprotective and to attenuate pro-inflammatory cytokine release post-TBI [139,140] and in other brain injury models [141]. We demonstrate a decrease in the pro-inflammatory cytokines KC/GRO and TNF-α in the cerebral cortex, as well as suppression in pro-inflammatory EV signaling pathways in female AD mice 7 days post-TBI, characterizing novel sex differences in inflammatory signaling in TBI with AD.

In this study, we also assessed the chronic expression profile of inflammasome components in TBI with AD to determine if acute changes in inflammasome expression found in our previous studies continued at longer time points after injury. First, we probed for levels of the pro-inflammatory cytokine IL-1β in the cerebral cortex and found that at 3 months post-injury, TBI levels were reduced to sham levels in the cerebral cortex of WT animals. However, the AD-TBI group still had significantly higher levels of IL-1β, indicating that the presence of AD genetic predisposition may have a role in post-TBI inflammatory pathology. When studying upstream inflammasome proteins, the same pattern was observed for NLRP3 and ASC protein expression, which was higher in the AD-TBI group when compared to the TBI-only group but not the AD-alone group. For NLRP1, we saw changes only between sham groups but not between injury comparisons. Caspase-1 was only significantly elevated in AD alone. When examining the expression of canonical inflammasome components in the hippocampus, no significant differences were seen between injury or genotype groups in ASC or caspase-1. Limited inflammasome activation in the hippocampus could be attributed to the severity of injury, which does not often involve the hippocampus in severity, or to a peak in signaling outside of the time points assessed. Further work will examine the inflammasome time course of activation in TBI with AD.

Since we have shown that there is elevation in non-canonical NLRP1, caspase-8, ASC inflammasome in the brain as a result of aging [76], we then examined whether components of the non-canonical inflammasome, caspases-8 and -11, were elevated in the AD-TBI group when compared to the other groups [18,19]. Non-canonical inflammasome pathways are often induced by LPS binding and often involve a more direct activation through the utilization of the caspase as sensor and effector, whereas canonical activation follows a standard binding of a DAMP and/or PAMP to a toll-like receptor, or the occurrence of a homeostasis-altering molecular process (HAMP), all of which lead to inflammasome sensor activation that leads to the release of pro-inflammatory cytokines [19,20]. In the current study, we detected increased caspase-8 expression within the AD-TBI group, indicative of a shift to a non-canonical inflammasome paradigm at the chronic stage in the AD group. Moreover, in prior assessments of caspase-8 activity in pre-clinical models of AD, reports indicate increased expression of caspase-8 in the cerebrum, which plays a role in Aβ seeding and microgliosis [142,143]. Furthermore, in humans with AD, caspase-8 has been shown to be expressed in hippocampal astrocytes [144], and caspase-8 signaling also plays a role in cell death and poor functional outcomes after TBI [145], with other inflammasome components also contributing to inflammation and pathological changes [146,147,148]. Therefore, we provide evidence of increased expression of the non-canonical inflammasome modulator caspase-8 in AD mice, and due to the increase in astrocyte activation denoted in our GFAP results, it is possible that astrocyte-mediated caspase-8 signaling in AD is leading to increased inflammasome component expression downstream of caspase-8 in our study’s time point.

The literature demonstrates that both TBI and AD alone lead to alterations in cortical and hippocampal volume. In AD mouse models, APPtg mice have more dramatic cortical thinning across life in comparison to WT mice [149]. Within the mouse model that we utilized in our study, there is evidence that there is a similar decreased hippocampal volume in mice starting at 5 weeks of age, with a trend of overall decreasing hippocampal volume in 3xTg clearly apparent [150,151]. These studies also pinpoint regional differences in areas such as the piriform cortex. In instances of TBI, we also see decreases in cortical volume after injury in murine models, though in moderate injuries, hippocampal atrophy is not often seen [75,152,153,154,155]. Due to the extent of past evidence, we sought to determine differences in volume between AD-TBI and WT-TBI groups. We normalized to total brain volume to account for volumetric differences inherent to each genetic strain and to AD pathology. Histologically, AD-TBI mice had evidence of cerebrocortical and hippocampal atrophy at greater levels than WT-TBI controls. In both TBI and AD, the loss of cerebral volume corresponds to worsening cognitive changes [156,157]. Thus, the increased cortical and hippocampal atrophy seen in TBI with AD is indicative of concerning pathological changes that are worth examining further. Future work replicating correlational analyses done in clinical populations on translational models of TBI with AD can provide insight into how brain volume changes can be predicted by accessible biomarkers.

Multiple studies have established the importance of NfL as a biomarker for neural degeneration after brain injury. Blood concentrations of NfL in TBI patients have been shown to be elevated 5 years after injury [158] and to correlate with white matter damage and other clinical outcomes [159,160]. Thus, in our study, we sought to determine the effect of AD comorbidity on post-TBI NfL levels, as AD has been shown to increase NfL [161]. The previous literature has demonstrated that AD-TBI mice have increased NfL levels up to 2-days post-injury [162]. We found that, even at 3 months post-TBI, the WT and AD groups had remarkably elevated NfL levels in the cortex when compared to sham controls, indicative of axonal damage in these groups.

Another important pathological marker is GFAP, an indicator of astrocyte activation. Serum levels of GFAP can distinguish patients who have undergone a TBI and can discriminate between those who need surgical intervention or who have intracranial lesions [163]. Studies have also shown that blood levels of GFAP can predict outcome and death after TBI [164,165], so much so that the FDA approved its use in blood testing to evaluate TBI patients [166]. Previous pre-clinical studies have shown that GFAP also increases acutely post-TBI [167,168]. In our study, we found a significant elevation of GFAP expression in the cerebral cortex in the AD-TBI group 3 months after TBI, indicating unresolved, chronic astrogliosis in this cohort. Thus, AD-TBI induces long-term astrogliosis, which may contribute to chronic post-injury difficulties.

Our results highlight pathological pathways that may underlie changes in AD and TBI between sexes. Further work on EV signaling should assess the mechanistic role that the identified pathways may play in AD and TBI sex differences, especially as EVs are now gaining attention as clinical tools. In TBI, EVs have been shown to have clinical diagnostic potential based upon the expression of certain miRNAs in the plasma of patients [169]. Moreover, brain-derived EVs also exhibit signaling signatures that can help mark the type of TBI and time of injury in pre-clinical and clinical settings [170]. In AD and TBI, EVs have been found to be potential biomarkers for degeneration based on their expression of Aβ and tau [171,172] and other markers, such as NfL [173]. In AD, EVs can differentiate between MCI and AD [174], demonstrating their capacity to inform clinical diagnostics about central nervous system signaling changes in a less invasive manner. Furthermore, therapeutically, inhibition of AD-associated EVs can improve synaptotoxicity and changes in long term potentiation in rodent models [175,176,177]. Thus, our results corroborate the importance of EVs in AD signaling and also shed some insights into how future therapeutics targeting EVs should keep in mind sex differences in their signaling.

In this study, we initially selected the 3xTg mouse model of AD pathology to study the different aspects of AD in which the inflammasome has been shown to play a role after TBI, namely, Aβ and tau pathology. This model allows the study of how the interaction between Aβ and tau affects inflammasome activation over time. Moreover, the present study continues our previous findings in the 3xTg model [21]. that assesses the interaction between TBI and AD acutely after TBI [178,179,180,181,182,183,184]. However, the 3xTg mouse model harbors familial genetic mutations that are not representative of the vast majority of AD patients in the population. The 3xTg mouse model is a triple transgenic mutant based upon the presence of the *PSEN1*, *APP*, and *MAPT* mutations. Thus, here we used the 3xTg AD model that reflects familial rather than sporadic AD, making it difficult to correlate findings of the present study in subjects who do not carry these genetic mutations. This model demonstrates clinically relevant hallmarks of AD, including extracellular Aβ deposits and phosphorylated tau tangles as well as cognitive impairment and microglial reactivity [185,186]. However, it is important to note that most cases of AD are of a sporadic nature [187], and the 3xTg model, being based upon a familial mutation of AD, is limited in its generalizability. Other mouse models that reflect the sporadic nature of AD and the influence of pertinent mutations such as *APOε* status post-TBI are currently underway.

Overall, we demonstrate that AD predisposition leads to chronic higher inflammasome component expression irrespective of injury status. Furthermore, the presence of AD predisposition also exacerbates hippocampal and cerebrocortical volume loss post-injury, as well as cellular astrocyte reactivity, after TBI. We also report that EV-mediated regulators and inflammatory pathways differ between male and female AD-TBI mice, specifically with females presenting with comparatively decreased inflammatory signaling but increased AD-associated pathway regulation. In addition, sex differences in inflammatory and AD-associated signaling provide further context for understanding mechanisms underlying varying outcomes between sexes. In conclusion, we have shown a chronic timeline of inflammatory pathology in AD mice, and we have documented sex differences to further understand both conditions. Thus, this study sheds light on the pathological interactions between AD and TBI.

## Data Availability

The raw data supporting the conclusions of this article will be made available by the authors, without undue reservation.

## Supplementary Materials

The following supporting information can be downloaded at: https://www.mdpi.com/article/10.3390/cells15181658/s1, Figure S1: Inflammasome protein expression is not chronically elevated in WT nor AD mice in the hippocampus 3 months post-TBI; Figure S2: Male and female AD-TBI mice do not present differences in Aβ species or GFAP 7 days post-injury. Figure S3: Immunoblotting Gels.

## References

[1] Dewan M.C., Rattani A., Gupta S., Baticulon R.E., Hung Y.-C., Punchak M., Agrawal A., Adeleye A.O., Shrime M.G., Rubiano A.M. (2019). Estimating the global incidence of traumatic brain injury. J. Neurosurg..

[2] Centers for Disease Control and Prevention (CDC) TBI Data. https://www.cdc.gov/traumatic-brain-injury/data-research/?CDC_AAref_Val=https://www.cdc.gov/traumaticbraininjury/data/index.html.

[3] Nichols E., Steinmetz J.D., Vollset S.E., Fukutaki K., Chalek J., Abd-Allah F., Abdoli A., Abualhasan A., Abu-Gharbieh E., Akram T.T. (2022). Estimation of the global prevalence of dementia in 2019 and forecasted prevalence in 2050: An analysis for the Global Burden of Disease Study 2019. Lancet Public Health.

[4] Jarrahi A., Braun M., Ahluwalia M., Gupta R.V., Wilson M., Munie S., Ahluwalia P., Vender J.R., Vale F.L., Dhandapani K.M. (2020). Revisiting Traumatic Brain Injury: From Molecular Mechanisms to Therapeutic Interventions. Biomedicines.

[5] de Macedo Filho L., Figueredo L.F., Villegas-Gomez G.A., Arthur M., Pedraza-Ciro M.C., Martins H., Kanawati Neto J., Hawryluk G.J., Amorim R.L.O. (2024). Pathophysiology-Based Management of Secondary Injuries and Insults in TBI. Biomedicines.

[6] McKee C.A., Lukens J.R. (2016). Emerging Roles for the Immune System in Traumatic Brain Injury. Front. Immunol..

[7] Gadani S.P., Walsh J.T., Lukens J.R., Kipnis J. (2015). Dealing with Danger in the CNS: The Response of the Immune System to Injury. Neuron.

[8] Wang Y., Mandelkow E. (2016). Tau in physiology and pathology. Nat. Rev. Neurosci..

[9] Delport A., Hewer R. (2022). The amyloid precursor protein: A converging point in Alzheimer’s disease. Mol. Neurobiol..

[10] Dams-O’Connor K., Guetta G., Hahn-Ketter A.E., Fedor A. (2016). Traumatic brain injury as a risk factor for Alzheimer’s disease: Current knowledge and future directions. Neurodegener. Dis. Manag..

[11] Rubenstein R., Chang B., Yue J.K., Chiu A., Winkler E.A., Puccio A.M., Diaz-Arrastia R., Yuh E.L., Mukherjee P., Valadka A.B. (2017). Comparing Plasma Phospho Tau, Total Tau, and Phospho Tau–Total Tau Ratio as Acute and Chronic Traumatic Brain Injury Biomarkers. JAMA Neurol..

[12] Abu Hamdeh S., Waara E.R., Möller C., Söderberg L., Basun H., Alafuzoff I., Hillered L., Lannfelt L., Ingelsson M., Marklund N. (2018). Rapid amyloid-β oligomer and protofibril accumulation in traumatic brain injury. Brain Pathol..

[13] DeKosky S.T., Abrahamson E.E., Ciallella J.R., Paljug W.R., Wisniewski S.R., Clark R.S., Ikonomovic M.D. (2007). Association of increased cortical soluble aβ42 levels with diffuse plaques after severe brain injury in humans. Arch. Neurol..

[14] Scott G., Ramlackhansingh A.F., Edison P., Hellyer P., Cole J., Veronese M., Leech R., Greenwood R.J., Turkheimer F.E., Gentleman S.M. (2016). Amyloid pathology and axonal injury after brain trauma. Neurology.

[15] Barnett K.C., Li S., Liang K., Ting J.P. (2023). A 360° view of the inflammasome: Mechanisms of activation, cell death, and diseases. Cell.

[16] Van Opdenbosch N., Lamkanfi M. (2019). Caspases in Cell Death, Inflammation, and Disease. Immunity.

[17] Sharma D., Kanneganti T.D. (2016). The cell biology of inflammasomes: Mechanisms of inflammasome activation and regulation. J. Cell Biol..

[18] Monie T.P., Bryant C.E. (2015). Caspase-8 functions as a key mediator of inflammation and pro-IL-1β processing via both canonical and non-canonical pathways. Immunol. Rev..

[19] Kayagaki N., Warming S., Lamkanfi M., Walle L.V., Louie S., Dong J., Newton K., Qu Y., Liu J., Heldens S. (2011). Non-canonical inflammasome activation targets caspase-11. Nature.

[20] Downs K.P., Nguyen H., Dorfleutner A., Stehlik C. (2020). An overview of the non-canonical inflammasome. Mol. Asp. Med..

[21] Johnson N.H., Kerr N.A., de Rivero Vaccari J.P., Bramlett H.M., Keane R.W., Dietrich W.D. (2023). Genetic predisposition to Alzheimer’s disease alters inflammasome activity after traumatic brain injury. Transl. Res..

[22] Lee S.W., de Rivero Vaccari J.P., Truettner J.S., Dietrich W.D., Keane R.W. (2019). The role of microglial inflammasome activation in pyroptotic cell death following penetrating traumatic brain injury. J. Neuroinflamm..

[23] Kerr N., Lee S.W., Perez-Barcena J., Crespi C., Ibañez J., Bullock M.R., Dietrich W.D., Keane R.W., de Rivero Vaccari J.P. (2019). Inflammasome proteins as biomarkers of traumatic brain injury. PLoS ONE.

[24] Pérez-Bárcena J., Pilar J.R., Salazar O., Crespí C., Frontera G., Novo M.A., Guardiola M.B., Llompart-Pou J.A., Ibáñez J., de Rivero Vaccari J.P. (2022). Serum Caspase-1 as an Independent Prognostic Factor in Traumatic Brain Injured Patients. Neurocritical Care.

[25] Scott X.O., Stephens M.E., Desir M.C., Dietrich W.D., Keane R.W., de Rivero Vaccari J.P. (2020). The Inflammasome Adaptor Protein ASC in Mild Cognitive Impairment and Alzheimer’s Disease. Int. J. Mol. Sci..

[26] Kerr N.A., de Rivero Vaccari J.P., Umland O., Bullock M.R., Conner G.E., Dietrich W.D., Keane R.W. (2019). Human Lung Cell Pyroptosis Following Traumatic Brain Injury. Cells.

[27] Cyr B., Ranaldi E.d.l.R.M.C., Hadad R., Dietrich W.D., Keane R.W., de Rivero Vaccari J.P. (2024). Extracellular vesicles mediate inflammasome signaling in the brain and heart of Alzheimer’s disease mice. Front. Mol. Neurosci..

[28] Keane R.W., Hadad R., Scott X.O., Ranaldi E.d.l.R.M.C., Pérez-Bárcena J., de Rivero Vaccari J.P. (2023). Neural–Cardiac Inflammasome Axis After Traumatic Brain Injury. Pharmaceuticals.

[29] Liu X., Zhang L., Cao Y., Jia H., Li X., Li F., Zhang S., Zhang J. (2023). Neuroinflammation of traumatic brain injury: Roles of extracellular vesicles. Front. Immunol..

[30] Hazelton I., Yates A., Dale A., Roodselaar J., Akbar N., Ruitenberg M.J., Anthony D.C., Couch Y. (2018). Exacerbation of Acute Traumatic Brain Injury by Circulating Extracellular Vesicles. J. Neurotrauma.

[31] Cai Z., Wan C.Q., Liu Z. (2017). Astrocyte and Alzheimer’s Disease. J. Neurol..

[32] Gupte R., Brooks W., Vukas R., Pierce J., Harris J. (2019). Sex Differences in Traumatic Brain Injury: What We Know and What We Should Know. J. Neurotrauma.

[33] Sass D., Guedes V.A., Smith E.G., Vorn R., Devoto C., Edwards K.A., Mithani S., Hentig J., Lai C., Wagner C. (2021). Sex Differences in Behavioral Symptoms and the Levels of Circulating GFAP, Tau, and NfL in Patients with Traumatic Brain Injury. Front. Pharmacol..

[34] Rubin T.G., Lipton M.L. (2019). Sex Differences in Animal Models of Traumatic Brain Injury. J. Exp. Neurosci..

[35] Rauk Z., Jędrusik J., Walczak Z., Setkowicz Z. (2025). Sex differences in neuropathological response to traumatic brain injury: Increased neuronal loss and astrogliosis in females. Brain Struct. Funct..

[36] Stein D.G. (2015). Embracing failure: What the Phase III progesterone studies can teach about TBI clinical trials. Brain Inj..

[37] O’Connor C.A., Cernak I., Johnson F., Vink R. (2007). Effects of progesterone on neurologic and morphologic outcome following diffuse traumatic brain injury in rats. Exp. Neurol..

[38] Rocca W.A., Grossardt B.R., Shuster L.T. (2011). Oophorectomy, menopause, estrogen treatment, and cognitive aging: Clinical evidence for a window of opportunity. Brain Res..

[39] Gao H., Fu L.-Y., Mu H.-Y., Sang C. (2021). Estrogen and cerebral small vessel disease. Chin. Med. J..

[40] Wang X., Feng S., Deng Q., Wu C., Duan R., Yang L. (2025). The role of estrogen in Alzheimer’s disease pathogenesis and therapeutic potential in women. Mol. Cell. Biochem..

[41] Valencia-Olvera A.C., Weng J.M., Christensen A., LaDu M.J., Pike C.J. (2023). Role of estrogen in women’s Alzheimer’s disease risk as modified by APOE. J. Neuroendocrinol..

[42] Carroll J.C., Rosario E.R., Chang L., Stanczyk F.Z., Oddo S., LaFerla F.M., Pike C.J. (2007). Progesterone and Estrogen Regulate Alzheimer-Like Neuropathology in Female 3xTg-AD Mice. J. Neurosci..

[43] Abi-Ghanem C., Opiela A.K., Paul A.S., Comito M.L., Hao L., Martino G., Kyaw N.-R., Salinero A.E., Mansour F.M., Kelly R.D. (2025). Loss of ovarian hormones is detrimental in early disease stages of mouse models of Alzheimer’s disease and multi-etiology dementia. Biol. Sex Differ..

[44] Blaya M.O., Raval A.P., Bramlett H.M. (2022). Traumatic brain injury in women across lifespan. Neurobiol. Dis..

[45] Beam C.R., Kaneshiro C., Jang J.Y., Reynolds C.A., Pedersen N.L., Gatz M. (2018). Differences Between Women and Men in Incidence Rates of Dementia and Alzheimer’s Disease. J. Alzheimers Dis..

[46] Aggarwal N.T., Mielke M.M. (2023). Sex Differences in Alzheimer’s Disease. Neurol. Clin..

[47] Filon J.R., Intorcia A.J., Sue L.I., Vazquez Arreola E., Wilson J., Davis K.J., Sabbagh M.N., Belden C.M., Caselli R.J., Adler C.H. (2016). Gender Differences in Alzheimer Disease: Brain Atrophy, Histopathology Burden, and Cognition. J. Neuropathol. Exp. Neurol..

[48] Oveisgharan S., Arvanitakis Z., Yu L., Farfel J., Schneider J.A., Bennett D.A. (2018). Sex differences in Alzheimer’s disease and common neuropathologies of aging. Acta Neuropathol..

[49] Barnes L.L., Wilson R.S., Bienias J.L., Schneider J.A., Evans D.A., Bennett D.A. (2005). Sex differences in the clinical manifestations of Alzheimer disease pathology. Arch. Gen. Psychiatry.

[50] Lin K.A., Choudhury K.R., Rathakrishnan B.G., Marks D.M., Petrella J.R., Doraiswamy P.M. (2015). Marked gender differences in progression of mild cognitive impairment over 8 years. Alzheimers Dement. Transl. Res. Clin. Interv..

[51] Kokiko-Cochran O., Ransohoff L., Veenstra M., Lee S., Saber M., Sikora M., Teknipp R., Xu G., Bemiller S., Wilson G. (2016). Altered Neuroinflammation and Behavior after Traumatic Brain Injury in a Mouse Model of Alzheimer’s Disease. J. Neurotrauma.

[52] Billings L.M., Oddo S., Green K.N., McGaugh J.L., LaFerla F.M. (2005). Intraneuronal Abeta causes the onset of early Alzheimer’s disease-related cognitive deficits in transgenic mice. Neuron.

[53] Oddo S., Caccamo A., Shepherd J.D., Murphy M.P., Golde T.E., Kayed R., Metherate R., Mattson M.P., Akbari Y., LaFerla F.M. (2003). Triple-transgenic model of Alzheimer’s disease with plaques and tangles: Intracellular Abeta and synaptic dysfunction. Neuron.

[54] Kerr N.A., Bramlett H.M., Sanchez-Molano J., Higueras A.F., Walters W., de Rivero Vaccari J.P., Keane R.W., Dietrich W.D. (2025). Stool-derived extracellular vesicles increase inflammasome signaling and regulate the gut-brain axis after stroke in Alzheimer’s disease transgenic mice. Exp. Neurol..

[55] Kerr N.A., Choi J., Mohite S.Y., Singh P.K., Bramlett H.M., Lee J.K., Dietrich W.D. (2025). Single cell RNA sequencing after moderate traumatic brain injury: Effects of therapeutic hypothermia. J. Neuroinflamm..

[56] Kerr N.A., Sanchez J., O’Connor G., Watson B.D., Daunert S., Bramlett H.M., Dietrich W.D. (2022). Inflammasome-Regulated Pyroptotic Cell Death in Disruption of the Gut-Brain Axis After Stroke. Transl. Stroke Res..

[57] Osier N.D., Dixon C.E. (2016). The Controlled Cortical Impact Model: Applications, Considerations for Researchers, and Future Directions. Front. Neurol..

[58] Kerr N.A., de Rivero Vaccari J.P., Weaver C., Dietrich W.D., Ahmed T., Keane R.W. (2021). Enoxaparin Attenuates Acute Lung Injury and Inflammasome Activation After Traumatic Brain Injury. J. Neurotrauma.

[59] Tran H.T., Sanchez L., Esparza T.J., Brody D.L. (2011). Distinct temporal and anatomical distributions of amyloid-β and tau abnormalities following controlled cortical impact in transgenic mice. PLoS ONE.

[60] Cora M.C., Kooistra L., Travlos G. (2015). Vaginal Cytology of the Laboratory Rat and Mouse: Review and Criteria for the Staging of the Estrous Cycle Using Stained Vaginal Smears. Toxicol. Pathol..

[61] Byers S.L., Wiles M.V., Dunn S.L., Taft R.A. (2012). Mouse Estrous Cycle Identification Tool and Images. PLoS ONE.

[62] Rowe R.K., Harrison J.L., Thomas T.C., Pauly J.R., Adelson P.D., Lifshitz J. (2013). Using anesthetics and analgesics in experimental traumatic brain injury. Lab Anim..

[63] Cyr B., de Rivero Vaccari J.P. (2023). Methods to Study Inflammasome Activation in the Central Nervous System: Immunoblotting and Immunohistochemistry. Methods Mol. Biol..

[64] Yang Y., Boza-Serrano A., Dunning C.J.R., Clausen B.H., Lambertsen K.L., Deierborg T. (2018). Inflammation leads to distinct populations of extracellular vesicles from microglia. J. Neuroinflamm..

[65] Iliuk A., Wu X., Li L., Sun J., Hadisurya M., Boris R.S., Tao W.A. (2020). Plasma-Derived Extracellular Vesicle Phosphoproteomics through Chemical Affinity Purification. J. Proteome Res..

[66] Chen I.-H., Xue L., Hsu C.-C., Paez J.S.P., Pan L., Andaluz H., Wendt M.K., Iliuk A.B., Zhu J.-K., Tao W.A. (2017). Phosphoproteins in extracellular vesicles as candidate markers for breast cancer. Proc. Natl. Acad. Sci. USA.

[67] Wu X., Quan M., Hadisurya M., Hu J., Liu Y.K., Zhuang Y., Li L., Iliuk A.B., Yang J.J., Kuang S. (2024). Monitoring drug metabolic pathways through extracellular vesicles in mouse plasma. Proc. Natl. Acad. Sci. Nexus.

[68] Hadad R., Keane R.W., de Rivero Vaccari J.P. (2023). Inflammasome signaling proteins as biomarkers of COVID-19. Front. Immunol..

[69] Bramlett H.M., Dietrich W.D. (2002). Quantitative structural changes in white and gray matter 1 year following traumatic brain injury in rats. Acta Neuropathol..

[70] Cyr B., de Rivero Vaccari J.P. (2023). Sex Differences in the Inflammatory Profile in the Brain of Young and Aged Mice. Cells.

[71] Moutinho S. (2025). Women twice as likely to develop Alzheimer’s disease as men—But scientists do not know why. Nat. Med..

[72] Ng K., Mikulis D.J., Glazer J., Kabani N., Till C., Greenberg G., Thompson A., Lazinski D., Agid R., Colella B. (2008). Magnetic Resonance Imaging Evidence of Progression of Subacute Brain Atrophy in Moderate to Severe Traumatic Brain Injury. Arch. Phys. Med. Rehabil..

[73] MacKenzie J.D., Siddiqi F., Babb J.S., Bagley L.J., Mannon L.J., Sinson G.P., Grossman R.I. (2002). Brain Atrophy in Mild or Moderate Traumatic Brain Injury: A Longitudinal Quantitative Analysis. Am. J. Neuroradiol..

[74] Zhang Y.P., Cai J., Shields L.B.E., Liu N., Xu X.-M., Shields C.B. (2014). Traumatic Brain Injury Using Mouse Models. Transl. Stroke Res..

[75] Yu S., Kaneko Y., Bae E., Stahl C.E., Wang Y., van Loveren H., Sanberg P.R., Borlongan C.V. (2009). Severity of controlled cortical impact traumatic brain injury in rats and mice dictates degree of behavioral deficits. Brain Res..

[76] Cyr B., Hadad R., Keane R.W., de Rivero Vaccari J.P. (2022). The Role of Non-canonical and Canonical Inflammasomes in Inflammaging. Front. Mol. Neurosci..

[77] Gaetani L., Blennow K., Calabresi P., Di Filippo M., Parnetti L., Zetterberg H. (2019). Neurofilament light chain as a biomarker in neurological disorders. J. Neurol. Neurosurg. Psychiatry.

[78] Burda J.E., Bernstein A.M., Sofroniew M.V. (2016). Astrocyte roles in traumatic brain injury. Exp. Neurol..

[79] de Rivero Vaccari J.P., Lotocki G., Alonso O.F., Bramlett H.M., Dietrich W.D., Keane R.W. (2009). Therapeutic neutralization of the NLRP1 inflammasome reduces the innate immune response and improves histopathology after traumatic brain injury. J. Cereb. Blood Flow Metab..

[80] Johnson N.H., Hadad R., Taylor R.R., Pilar J.R., Salazar O., Llompart-Pou J.A., Dietrich W.D., Keane R.W., Pérez-Bárcena J., de Rivero Vaccari J.P. (2022). Inflammatory Biomarkers of Traumatic Brain Injury. Pharmaceuticals.

[81] Sivanandam T.M., Thakur M.K. (2012). Traumatic brain injury: A risk factor for Alzheimer’s disease. Neurosci. Biobehav. Rev..

[82] Johnson N.H., de Rivero Vaccari J.P., Bramlett H.M., Keane R.W., Dietrich W.D. (2023). Inflammasome activation in traumatic brain injury and Alzheimer’s disease. Transl. Res..

[83] Mohamed A.Z., Nestor P.J., Cumming P., Nasrallah F.A. (2022). Traumatic brain injury fast-forwards Alzheimer’s pathology: Evidence from amyloid positron emission tomorgraphy imaging. J. Neurol..

[84] Corps K.N., Roth T.L., McGavern D.B. (2015). Inflammation and Neuroprotection in Traumatic Brain Injury. JAMA Neurol..

[85] O’Brien W.T., Pham L., Symons G.F., Monif M., Shultz S.R., McDonald S.J. (2020). The NLRP3 inflammasome in traumatic brain injury: Potential as a biomarker and therapeutic target. J. Neuroinflamm..

[86] Tan M.-S., Yu J.-T., Jiang T., Zhu X.-C., Tan L. (2013). The NLRP3 Inflammasome in Alzheimer’s Disease. Mol. Neurobiol..

[87] Heppner F.L., Ransohoff R.M., Becher B. (2015). Immune attack: The role of inflammation in Alzheimer disease. Nat. Rev. Neurosci..

[88] Bramlett H.M., Kraydieh S., Green E.J., Dietrich W.D. (1997). Temporal and Regional Patterns of Axonal Damage Following Traumatic Brain Injury: A Beta-Amyloid Precursor Protein Immunocytochemical Study in Rats. J. Neuropathol. Exp. Neurol..

[89] de Rivero Vaccari J.P., Davis D.A., Sawaya A.P., Garamszegi S.P., Sun X., Barreda A., Bramlett H.M., Gultekin S.H., Dietrich W.D., Keane R.W. (2026). Increased expression of inflammasome signaling genes and proteins in selective brain regions in the intermediate stage of Alzheimer’s disease. Brain Pathol..

[90] Vontell R.T., de Rivero Vaccari J.P., Sun X., Gultekin S.H., Bramlett H.M., Dietrich W.D., Keane R.W. (2023). Identification of inflammasome signaling proteins in neurons and microglia in early and intermediate stages of Alzheimer’s disease. Brain Pathol..

[91] Vontell R.T., Gober R., Dallmeier J., Brzostowicki D., Barreda A., Blennow K., Zetterberg H., Kvartsberg H., Gultekin S.H., de Rivero Vaccari J.P. (2024). Association of region-specific hippocampal reduction of neurogranin with inflammasome proteins in post mortem brains of Alzheimer’s disease. Alzheimers Dement. Transl. Res. Clin. Interv..

[92] Ge X., Li W., Huang S., Yin Z., Xu X., Chen F., Kong X., Wang H., Zhang J., Lei P. (2018). The pathological role of NLRs and AIM2 inflammasome-mediated pyroptosis in damaged blood-brain barrier after traumatic brain injury. Brain Res..

[93] Ma J., Xiao W., Wang J., Wu J., Ren J., Hou J., Gu J., Fan K., Yu B. (2016). Propofol Inhibits NLRP3 Inflammasome and Attenuates Blast-Induced Traumatic Brain Injury in Rats. Inflammation.

[94] Saresella M., La Rosa F., Piancone F., Zoppis M., Marventano I., Calabrese E., Rainone V., Nemni R., Mancuso R., Clerici M. (2016). The NLRP3 and NLRP1 inflammasomes are activated in Alzheimer’s disease. Mol. Neurodegener..

[95] Zhao Q., Zhang J., Li H., Li H., Xie F. (2023). Models of traumatic brain injury-highlights and drawbacks. Front. Neurol..

[96] Najem D., Rennie K., Ribecco-Lutkiewicz M., Ly D., Haukenfrers J., Liu Q., Nzau M., Fraser D.D., Bani-Yaghoub M. (2018). Traumatic brain injury: Classification, models, and markers. Biochem. Cell Biol..

[97] Xiong Y., Mahmood A., Chopp M. (2013). Animal models of traumatic brain injury. Nat. Rev. Neurosci..

[98] Huang Y., Feng J., Xu J., Dong L., Su W., Li B., Witwer K.W., Zheng L. (2025). Associations of age and sex with characteristics of extracellular vesicles and protein-enriched fractions of blood plasma. Aging Cell.

[99] Elsherbini A., Zhu Z., Quadri Z., Crivelli S.M., Ren X., Vekaria H.J., Tripathi P., Zhang L., Zhi W., Bieberich E. (2023). Novel Isolation Method Reveals Sex-Specific Composition and Neurotoxicity of Small Extracellular Vesicles in a Mouse Model of Alzheimer’s Disease. Cells.

[100] Sawaya A.P., Stone R.C., Brooks S.R., Pastar I., Jozic I., Hasneen K., O’Neill K., Mehdizadeh S., Head C.R., Strbo N. (2020). Deregulated immune cell recruitment orchestrated by FOXM1 impairs human diabetic wound healing. Nat. Commun..

[101] Bodart-Santos V., Pinheiro L.S., da Silva-Junior A.J., Froza R.L., Ahrens R., Gonçalves R.A., Andrade M.M., Chen Y., Alcantara C.d.L., Grinberg L.T. (2023). Alzheimer’s disease brain-derived extracellular vesicles reveal altered synapse-related proteome and induce cognitive impairment in mice. Alzheimers Dement..

[102] Muraoka S., Jedrychowski M.P., Iwahara N., Abdullah M., Onos K.D., Keezer K.J., Hu J., Ikezu S., Howell G.R., Gygi S.P. (2021). Enrichment of Neurodegenerative Microglia Signature in Brain-Derived Extracellular Vesicles Isolated from Alzheimer’s Disease Mouse Models. J. Proteome Res..

[103] Anastasi F., Greco F., Dilillo M., Vannini E., Cappello V., Baroncelli L., Costa M., Gemmi M., Caleo M., McDonnell L.A. (2020). Proteomics analysis of serum small extracellular vesicles for the longitudinal study of a glioblastoma multiforme mouse model. Sci. Rep..

[104] Hurwitz S.N., Sun L., Cole K.Y., Ford C.R., Olcese J.M., Meckes D.G. (2018). An optimized method for enrichment of whole brain-derived extracellular vesicles reveals insight into neurodegenerative processes in a mouse model of Alzheimer’s disease. J. Neurosci. Methods.

[105] Kang J., Rivest S. (2012). Lipid Metabolism and Neuroinflammation in Alzheimer’s Disease: A Role for Liver X Receptors. Endocr. Rev..

[106] Litvinchuk A., Suh J.H., Guo J.L., Lin K., Davis S.S., Bien-Ly N., Tycksen E., Tabor G.T., Remolina Serrano J., Manis M. (2024). Amelioration of Tau and ApoE4-linked glial lipid accumulation and neurodegeneration with an LXR agonist. Neuron.

[107] Zhang S., Yu Q., Li Z., Zhao Y., Sun Y. (2024). Protein neddylation and its role in health and diseases. Signal Transduct. Target. Ther..

[108] Chen Y., Neve R.L., Liu H. (2012). Neddylation dysfunction in Alzheimer’s disease. J. Cell. Mol. Med..

[109] Yu H., Luo H., Chang L., Wang S., Geng X., Kang L., Zhong Y., Cao Y., Wang R., Yang X. (2022). The NEDD8-activating enzyme inhibitor MLN4924 reduces ischemic brain injury in mice. Proc. Natl. Acad. Sci. USA.

[110] Afify R., Lipsius K., Wyatt-Johnson S.J., Brutkiewicz R.R. (2024). Myeloid antigen-presenting cells in neurodegenerative diseases: A focus on classical and non-classical MHC molecules. Front. Neurosci..

[111] Thome J.G., Reeder E.L., Collins S.M., Gopalan P., Robson M.J. (2020). Contributions of Interleukin-1 Receptor Signaling in Traumatic Brain Injury. Front. Behav. Neurosci..

[112] Özen I., Clausen F., Flygt J., Marklund N., Paul G. (2024). Neutralization of Interleukin 1-beta is associated with preservation of thalamic capillaries after experimental traumatic brain injury. Front. Neurol..

[113] Shaftel S.S., Griffin W.S., O’Banion M.K. (2008). The role of interleukin-1 in neuroinflammation and Alzheimer disease: An evolving perspective. J. Neuroinflamm..

[114] Liu T., Li X. (2025). The Crosstalk Between Protective and Detrimental Interleukin (IL)-1 Family of Cytokines in Alzheimer’s Disease. J. Biochem. Mol. Toxicol..

[115] Pietronigro E., Zenaro E., Constantin G. (2016). Imaging of Leukocyte Trafficking in Alzheimer’s Disease. Front. Immunol..

[116] Pietronigro E., Zenaro E., Bianca V.D., Dusi S., Terrabuio E., Iannoto G., Slanzi A., Ghasemi S., Nagarajan R., Piacentino G. (2019). Blockade of α4 integrins reduces leukocyte–endothelial interactions in cerebral vessels and improves memory in a mouse model of Alzheimer’s disease. Sci. Rep..

[117] Hubbard W.B., Dong J.-f., Cruz M.A., Rumbaut R.E. (2021). Links between thrombosis and inflammation in traumatic brain injury. Thromb. Res..

[118] Belloy M.E., Napolioni V., Greicius M.D. (2019). A Quarter Century of APOE and Alzheimer’s Disease: Progress to Date and the Path Forward. Neuron.

[119] Ungar L., Altmann A., Greicius M.D. (2014). Apolipoprotein E, gender, and Alzheimer’s disease: An overlooked, but potent and promising interaction. Brain Imaging Behav..

[120] Xu X., Kwon J., Yan R., Apio C., Song S., Heo G., Yang Q., Timsina J., Liu M., Budde J. (2025). Sex Differences in Apolipoprotein E and Alzheimer Disease Pathology Across Ancestries. JAMA Netw. Open.

[121] Ponsford J., McLaren A., Schönberger M., Burke R., Rudzki D., Olver J., Ponsford M. (2011). The association between apolipoprotein E and traumatic brain injury severity and functional outcome in a rehabilitation sample. J. Neurotrauma.

[122] Mosconi L., Berti V., Quinn C., McHugh P., Petrongolo G., Varsavsky I., Osorio R.S., Pupi A., Vallabhajosula S., Isaacson R.S. (2017). Sex differences in Alzheimer risk. Neurology.

[123] Hansson O., Lehmann S., Otto M., Zetterberg H., Lewczuk P. (2019). Advantages and disadvantages of the use of the CSF Amyloid β (Aβ) 42/40 ratio in the diagnosis of Alzheimer’s Disease. Alzheimers Res. Ther..

[124] Dennison J.L., Ricciardi N.R., Lohse I., Volmar C.-H., Wahlestedt C. (2021). Sexual Dimorphism in the 3xTg-AD Mouse Model and Its Impact on Pre-Clinical Research. J. Alzheimers Dis..

[125] Villapol S., Loane D.J., Burns M.P. (2017). Sexual dimorphism in the inflammatory response to traumatic brain injury. Glia.

[126] Doran S.J., Ritzel R.M., Glaser E.P., Henry R.J., Faden A.I., Loane D.J. (2019). Sex Differences in Acute Neuroinflammation After Experimental Traumatic Brain Injury Are Mediated by Infiltrating Myeloid Cells. J. Neurotrauma.

[127] Royal T., Ahluwalia P., Gulhane M., Salles E.L., Gaur P., Ahluwalia M., Randhawa T., Sunil S., Budim S., Akter K. (2026). Neuroinflammatory and functional outcomes after TBI are sex-dependent: Lessons from estrous-phase stratified female mice. Neurochem. Int..

[128] Barber A.J., del Genio C.L., Swain A.B., Pizzi E.M., Watson S.C., Tapiavala V.N., Zanazzi G.J., Gaur A.B. (2024). Age, sex and Alzheimer’s disease: A longitudinal study of 3xTg-AD mice reveals sex-specific disease trajectories and inflammatory responses mirrored in postmortem brains from Alzheimer’s patients. Alzheimers Res. Ther..

[129] Johnson E.A., Dao T.L., Guignet M.A., Geddes C.E., Koemeter-Cox A.I., Kan R.K. (2011). Increased expression of the chemokines CXCL1 and MIP-1α by resident brain cells precedes neutrophil infiltration in the brain following prolonged soman-induced status epilepticus in rats. J. Neuroinflamm..

[130] Chen Y., Wang Y., Xu J., Hou T., Zhu J., Jiang Y., Sun L., Huang C., Sun L., Liu S. (2023). Multiplex Assessment of Serum Chemokines CCL2, CCL5, CXCL1, CXCL10, and CXCL13 Following Traumatic Brain Injury. Inflammation.

[131] Schädlich I.S., Vienhues J.H., Jander A., Piepke M., Magnus T., Lambertsen K.L., Clausen B.H., Gelderblom M. (2022). Interleukin-1 Mediates Ischemic Brain Injury via Induction of IL-17A in γδ T Cells and CXCL1 in Astrocytes. NeuroMolecular Med..

[132] Huang X., Guo M., Zhang Y., Xie J., Huang R., Zuo Z., Saw P.E., Cao M. (2023). Microglial IL-1RA ameliorates brain injury after ischemic stroke by inhibiting astrocytic CXCL1-mediated neutrophil recruitment and microvessel occlusion. Glia.

[133] Berrrrpohl D., You Z., Lo E.H., Kim H.-H., Whalen M.J. (2007). TNF Alpha and Fas Mediate Tissue Damage and Functional Outcome after Traumatic Brain Injury in Mice. J. Cereb. Blood Flow Metab..

[134] Hayakata T., Shiozaki T., Tasaki O., Ikegawa H., Inoue Y., Toshiyuki F., Hosotubo H., Kieko F., Yamashita T., Tanaka H. (2004). Changes in CSF S100B and cytokine concentrations in early-phase severe traumatic brain injury. Shock.

[135] Yan E.B., Satgunaseelan L., Paul E., Bye N., Nguyen P., Agyapomaa D., Kossmann T., Rosenfeld J.V., Morganti-Kossmann M.C. (2013). Post-Traumatic Hypoxia Is Associated with Prolonged Cerebral Cytokine Production, Higher Serum Biomarker Levels, and Poor Outcome in Patients with Severe Traumatic Brain Injury. J. Neurotrauma.

[136] Scherbel U., Raghupathi R., Nakamura M., Saatman K.E., Trojanowski J.Q., Neugebauer E., Marino M.W., McIntosh T.K. (1999). Differential acute and chronic responses of tumor necrosis factor-deficient mice to experimental brain injury. Proc. Natl. Acad. Sci. USA.

[137] Robison L.S., Gannon O.J., Salinero A.E., Abi-Ghanem C., Kelly R.D., Riccio D.A., Mansour F.M., Zuloaga K.L. (2023). Sex differences in metabolic phenotype and hypothalamic inflammation in the 3xTg-AD mouse model of Alzheimer’s disease. Biol. Sex Differ..

[138] Ajayi A.F., Akhigbe R.E. (2020). Staging of the estrous cycle and induction of estrus in experimental rodents: An update. Fertil. Res. Pract..

[139] Pan D.S., Liu W.G., Yang X.F., Cao F. (2007). Inhibitory effect of progesterone on inflammatory factors after experimental traumatic brain injury. Biomed. Environ. Sci..

[140] Zhou Z., Li Y., Peng R., Shi M., Gao W., Lei P., Zhang J. (2024). Progesterone induces neuroprotection associated with immune/inflammatory modulation in experimental traumatic brain injury. NeuroReport.

[141] Aggarwal R., Medhi B., Pathak A., Dhawan V., Chakrabarti A. (2008). Neuroprotective effect of progesterone on acute phase changes induced by partial global cerebral ischaemia in mice. J. Pharm. Pharmacol..

[142] Huang Y., Li X., Luo G., Wang J., Li R., Zhou C., Wan T., Yang F. (2022). Pyroptosis as a candidate therapeutic target for Alzheimer’s disease. Front. Aging Neurosci..

[143] Kumar S., Budhathoki S., Oliveira C.B., Kahle A.D., Calhan O.Y., Lukens J.R., Deppmann C.D. (2023). Role of the caspase-8/RIPK3 axis in Alzheimer’s disease pathogenesis and Aβ-induced NLRP3 inflammasome activation. JCI Insight.

[144] Moonen S., Koper M.J., Van Schoor E., Schaeverbeke J.M., Vandenberghe R., von Arnim C.A.F., Tousseyn T., De Strooper B., Thal D.R. (2023). Pyroptosis in Alzheimer’s disease: Cell type-specific activation in microglia, astrocytes and neurons. Acta Neuropathol..

[145] Krajewska M., You Z., Rong J., Kress C., Huang X., Yang J., Kyoda T., Leyva R., Banares S., Hu Y. (2011). Neuronal Deletion of Caspase 8 Protects Against Brain Injury in Mouse Models of Controlled Cortical Impact and Kainic Acid-Induced Excitotoxicity. PLoS ONE.

[146] Ozen I., Ruscher K., Nilsson R., Flygt J., Clausen F., Marklund N. (2020). Interleukin-1 Beta Neutralization Attenuates Traumatic Brain Injury-Induced Microglia Activation and Neuronal Changes in the Globus Pallidus. Int. J. Mol. Sci..

[147] Chakraborty R., Tabassum H., Parvez S. (2023). NLRP3 inflammasome in traumatic brain injury: Its implication in the disease pathophysiology and potential as a therapeutic target. Life Sci..

[148] Liu W., Chen Y., Meng J., Wu M., Bi F., Chang C., Li H., Zhang L. (2018). Ablation of caspase-1 protects against TBI-induced pyroptosis in vitro and in vivo. J. Neuroinflamm..

[149] Hébert F., Grand’Maison M., Ho M.-K., Lerch J.P., Hamel E., Bedell B.J. (2013). Cortical atrophy and hypoperfusion in a transgenic mouse model of Alzheimer’s disease. Neurobiol. Aging.

[150] Chiquita S., Ribeiro M., Castelhano J., Oliveira F., Sereno J., Batista M., Abrunhosa A., Rodrigues-Neves A.C., Carecho R., Baptista F. (2019). A longitudinal multimodal in vivo molecular imaging study of the 3xTg-AD mouse model shows progressive early hippocampal and taurine loss. Hum. Mol. Genet..

[151] Kong V., Devenyi G.A., Gallino D., Ayranci G., Germann J., Rollins C., Chakravarty M.M. (2018). Early-in-life neuroanatomical and behavioural trajectories in a triple transgenic model of Alzheimer’s disease. Brain Struct. Funct..

[152] Niesman I.R., Schilling J.M., Shapiro L.A., Kellerhals S.E., Bonds J.A., Kleschevnikov A.M., Cui W., Voong A., Krajewski S., Ali S.S. (2014). Traumatic brain injury enhances neuroinflammation and lesion volume in caveolin deficient mice. J. Neuroinflamm..

[153] Siebold L., Obenaus A., Goyal R. (2018). Criteria to define mild, moderate, and severe traumatic brain injury in the mouse controlled cortical impact model. Exp. Neurol..

[154] Raible D.J., Frey L.C., Del Angel Y.C., Carlsen J., Hund D., Russek S.J., Smith B., Brooks-Kayal A.R. (2015). JAK/STAT pathway regulation of GABAA receptor expression after differing severities of experimental TBI. Exp. Neurol..

[155] Budinich C.S., Tucker L.B., Lowe D., Rosenberger J.G., McCabe J.T. (2013). Short and long-term motor and behavioral effects of diazoxide and dimethyl sulfoxide administration in the mouse after traumatic brain injury. Pharmacol. Biochem. Behav..

[156] Bilello M., Doshi J., Nabavizadeh S.A., Toledo J.B., Erus G., Xie S.X., Trojanowski J.Q., Han X., Davatzikos C. (2015). Correlating Cognitive Decline with White Matter Lesion and Brain Atrophy Magnetic Resonance Imaging Measurements in Alzheimer’s Disease. J. Alzheimers Dis..

[157] Tate D.F., Khedraki R., Neeley E.S., Ryser D.K., Bigler E.D. (2011). Cerebral Volume Loss, Cognitive Deficit, and Neuropsychological Performance: Comparative Measures of Brain Atrophy: II. Traumatic Brain Injury. J. Int. Neuropsychol. Soc..

[158] Shahim P., Politis A., van der Merwe A., Moore B., Ekanayake V., Lippa S.M., Chou Y.-Y., Pham D.L., Butman J.A., Diaz-Arrastia R. (2020). Time course and diagnostic utility of NfL, tau, GFAP, and UCH-L1 in subacute and chronic TBI. Neurology.

[159] Graham N.S.N., Zimmerman K.A., Moro F., Heslegrave A., Maillard S.A., Bernini A., Miroz J.-P., Donat C.K., Lopez M.Y., Bourke N. (2021). Axonal marker neurofilament light predicts long-term outcomes and progressive neurodegeneration after traumatic brain injury. Sci. Transl. Med..

[160] Shahim P., Politis A., van der Merwe A., Moore B., Chou Y.-Y., Pham D.L., Butman J.A., Diaz-Arrastia R., Gill J.M., Brody D.L. (2020). Neurofilament light as a biomarker in traumatic brain injury. Neurology.

[161] Bacioglu M., Maia L.F., Preische O., Schelle J., Apel A., Kaeser S.A., Schweighauser M., Eninger T., Lambert M., Pilotto A. (2016). Neurofilament Light Chain in Blood and CSF as Marker of Disease Progression in Mouse Models and in Neurodegenerative Diseases. Neuron.

[162] Cheng W.H., Stukas S., Martens K.M., Namjoshi D.R., Button E.B., Wilkinson A., Bashir A., Robert J., Cripton P.A., Wellington C.L. (2018). Age at injury and genotype modify acute inflammatory and neurofilament-light responses to mild CHIMERA traumatic brain injury in wild-type and APP/PS1 mice. Exp. Neurol..

[163] Papa L., Brophy G.M., Welch R.D., Lewis L.M., Braga C.F., Tan C.N., Ameli N.J., Lopez M.A., Haeussler C.A., Mendez Giordano D.I. (2016). Time Course and Diagnostic Accuracy of Glial and Neuronal Blood Biomarkers GFAP and UCH-L1 in a Large Cohort of Trauma Patients With and Without Mild Traumatic Brain Injury. JAMA Neurol..

[164] Vos P.E., Jacobs B., Andriessen T.M., Lamers K.J., Borm G.F., Beems T., Edwards M., Rosmalen C.F., Vissers J.L. (2010). GFAP and S100B are biomarkers of traumatic brain injury: An observational cohort study. Neurology.

[165] Nylén K., Ost M., Csajbok L.Z., Nilsson I., Blennow K., Nellgård B., Rosengren L. (2006). Increased serum-GFAP in patients with severe traumatic brain injury is related to outcome. J. Neurol. Sci..

[166] U.S. Food and Drug Administration (2018). FDA Authorizes Marketing of First Blood Test to Aid in the Evaluation of Concussion in Adults.

[167] Kane M.J., Angoa-Pérez M., Briggs D.I., Viano D.C., Kreipke C.W., Kuhn D.M. (2012). A mouse model of human repetitive mild traumatic brain injury. J. Neurosci. Methods.

[168] Huang X.-j., Glushakova O., Mondello S., Van K., Hayes R.L., Lyeth B.G. (2015). Acute Temporal Profiles of Serum Levels of UCH-L1 and GFAP and Relationships to Neuronal and Astroglial Pathology Following Traumatic Brain Injury in Rats. J. Neurotrauma.

[169] Ghai V., Fallen S., Baxter D., Scherler K., Kim T.-K., Zhou Y., Meabon J.S., Logsdon A.F., Banks W.A., Schindler A.G. (2020). Alterations in plasma microRNA and protein levels in war veterans with chronic mild traumatic brain injury. J. Neurotrauma.

[170] Ko J., Hemphill M., Yang Z., Beard K., Sewell E., Shallcross J., Schweizer M., Sandsmark D.K., Diaz-Arrastia R., Kim J. (2020). Multi-Dimensional Mapping of Brain-Derived Extracellular Vesicle MicroRNA Biomarker for Traumatic Brain Injury Diagnostics. J. Neurotrauma.

[171] Khan N.A., Asim M., El-Menyar A., Biswas K.H., Rizoli S., Al-Thani H. (2022). The evolving role of extracellular vesicles (exosomes) as biomarkers in traumatic brain injury: Clinical perspectives and therapeutic implications. Front. Aging Neurosci..

[172] Vaz M., Soares Martins T., Henriques A.G. (2022). Extracellular vesicles in the study of Alzheimer’s and Parkinson’s diseases: Methodologies applied from cells to biofluids. J. Neurochem..

[173] Guedes V.A., Lange R.T., Lippa S.M., Lai C., Greer K., Mithani S., Devoto C., Edwards K.A., Wagner C.L., Martin C.A. (2022). Extracellular vesicle neurofilament light is elevated within the first 12-months following traumatic brain injury in a U.S military population. Sci. Rep..

[174] Muraoka S., Jedrychowski M.P., Yanamandra K., Ikezu S., Gygi S.P., Ikezu T. (2020). Proteomic profiling of extracellular vesicles derived from cerebrospinal fluid of Alzheimer’s disease patients: A pilot study. Cells.

[175] Crivelli S.M., Quadri Z., Vekaria H.J., Zhu Z., Tripathi P., Elsherbini A., Zhang L., Sullivan P.G., Bieberich E. (2023). Inhibition of acid sphingomyelinase reduces reactive astrocyte secretion of mitotoxic extracellular vesicles and improves Alzheimer’s disease pathology in the 5xFAD mouse. Acta Neuropathol. Commun..

[176] Yu Y., Ma Z., Li T., Xiao W., Li Z. (2026). Neuronal Extracellular Vesicles Carrying APOE Downregulate Filament Actin Polymerization Signaling to Inhibit Synapse Formation in Alzheimer’s Disease. J. Extracell. Vesicles.

[177] Gabrielli M., Prada I., Joshi P., Falcicchia C., D’Arrigo G., Rutigliano G., Battocchio E., Zenatelli R., Tozzi F., Radeghieri A. (2022). Microglial large extracellular vesicles propagate early synaptic dysfunction in Alzheimer’s disease. Brain.

[178] Wu Z., Wang Z.-H., Liu X., Zhang Z., Gu X., Yu S.P., Keene C.D., Cheng L., Ye K. (2020). Traumatic brain injury triggers APP and Tau cleavage by delta-secretase, mediating Alzheimer’s disease pathology. Prog. Neurobiol..

[179] Green J., Liu Z., Timis S., Nelson C., Pedin A., Guo C., Wang X.-Y., Zhang S., Sun D. (2026). Deletion of NLRP3 gene blocks traumatic brain injury induced abnormal immune response in 3xTg AD mice. bioRxiv.

[180] Pybus A.F., Bitarafan S., Brothers R.O., Rohrer A., Khaitan A., Moctezuma F.R., Udeshi K., Davies B., Triplett S., Griffin M.N. (2024). Profiling the neuroimmune cascade in 3xTg-AD mice exposed to successive mild traumatic brain injuries. J. Neuroinflamm..

[181] Tran H.T., LaFerla F.M., Holtzman D.M., Brody D.L. (2011). Controlled Cortical Impact Traumatic Brain Injury in 3xTg-AD Mice Causes Acute Intra-Axonal Amyloid-β Accumulation and Independently Accelerates the Development of Tau Abnormalities. J. Neurosci..

[182] Bennett R.E., Esparza T.J., Lewis H.A., Kim E., Mac Donald C.L., Sullivan P.M., Brody D.L. (2013). Human Apolipoprotein E4 Worsens Acute Axonal Pathology but Not Amyloid-β Immunoreactivity After Traumatic Brain Injury in 3×TG-AD Mice. J. Neuropathol. Exp. Neurol..

[183] Barker C.C.H., Koza L.A., Almuhanna L., Trujillo K.R., Linseman D.A. (2026). Lack of effect of repetitive mild traumatic brain injury early in life on the neuropathological and behavioral hallmarks of Alzheimer’s disease in 3xTg-AD mice. J. Alzheimers Dis..

[184] Hua N., Minaeva O., Parsons D., Moncaster J.A., Demb E., Goldstein L.E. (2025). High-Resolution Diffusion-MRI Detects Degeneration in Hippocampal Subregions After Neurotrauma in 3xTg-AD Mice. Alzheimers Dement..

[185] Pattanayak A., Firdous S.M. (2026). The 3xTg-AD Mouse Model: A Comprehensive Tool for Understanding Alzheimer’s Disease. Cell. Mol. Neurobiol..

[186] Zhong M.Z., Peng T., Duarte M.L., Wang M., Cai D. (2024). Updates on mouse models of Alzheimer’s disease. Mol. Neurodegener..

[187] Thies W., Bleiler L., Alzheimer’s Association (2013). 2013 Alzheimer’s disease facts and figures. Alzheimers Dement..

