# Supplementary material for: Alzheimer’s Disease Leads to Chronic Pathological Changes and Inflammasome Signaling in an Animal Model of Traumatic Brain Injury
Source: Cells. 2026 Sep 14;15(18):1658. doi: 10.3390/cells15181658 (PMC13604984; doi:10.3390/cells15181658)

SUPPLEMENTARY MATERIAL

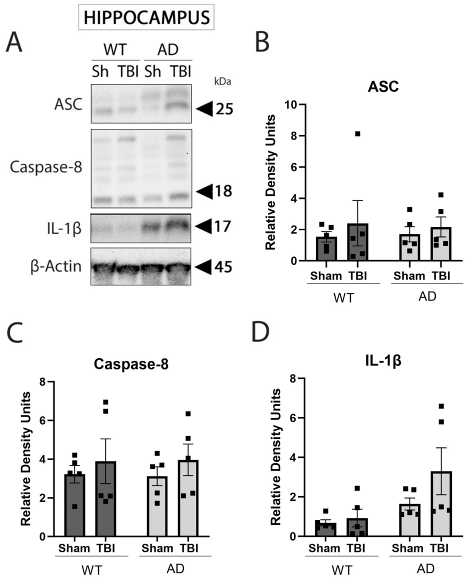

Figure S1. Inflammasome Protein Expression is not Chronically Elevated in WT nor AD Mice in the Hippocampus 3 Months Post-TBI. A) Representative immunoblots normalized to β-actin for inflammasome signaling protein expression in hippocampal lysates from AD and WT mice with or without moderate CCI. **B)** ASC protein expression levels did not differ between any of the experimental groups in the hippocampus at 3-months post-TBI. **C)** Caspase-8 protein expression was unchanged at 3-months post-TBI in the hippocampus of WT and AD mice. **D)** Densitometric analysis did not detect any significant changes in IL-1β protein expression in the hippocampus of WT nor AD mice 3-months post-TBI. Data shown as mean+/-SEM. A two-way ANOVA with Šidák's multiple comparisons test was used to compare groups. N = 5 per group with significant p values shown.

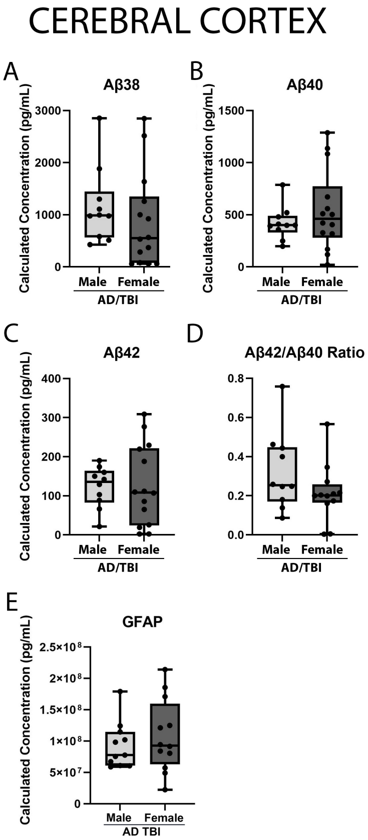

Figure S2. Male and Female AD-TBI Mice do not present Differences in Aβ Species or GFAP 7 Days Post-Injury. Cerebral cortical lysates of male and female AD mice do not have significant differences in Aβ-38 **(A)**, Aβ-40 **(B)**, nor Aβ-42 **(C). D)** There were also no significant differences in the Aβ-42/Aβ-40 ratio between sexes of AD-TBI mice. **E)** There were also no sex differences in GFAP expression in the cerebral cortex of AD-TBI male and female mice 7-days post-TBI. Data shown as mean+/-SEM. Data were analyzed using a Mann Whitney test between groups. N = 14/group.

Figure S3: Immunoblotting Gels

Cerebral Cortex
(WT-SH, WT-TBI, AD-SH, AD-TBI) x 5

IL-1β

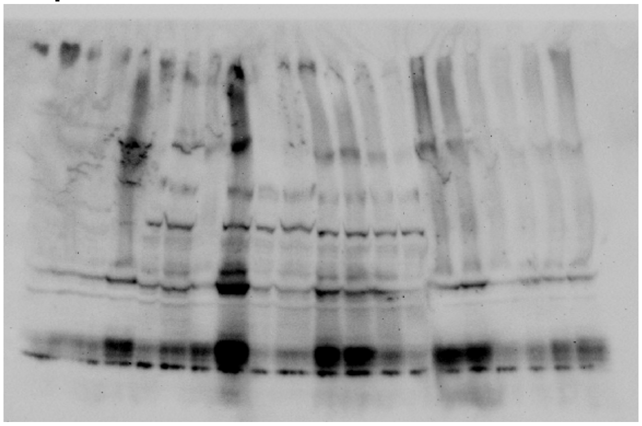

NLRP3

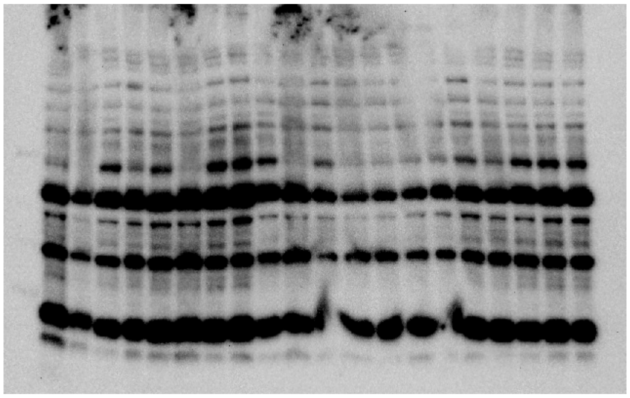

NLRP1

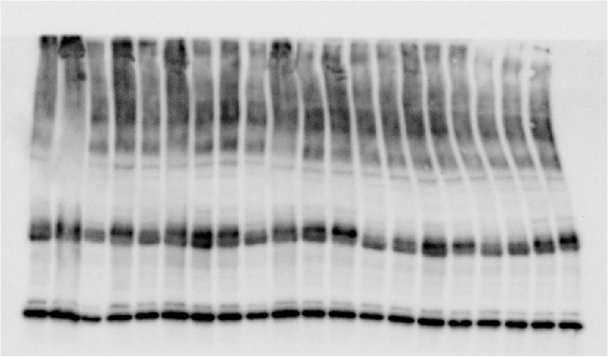

ASC

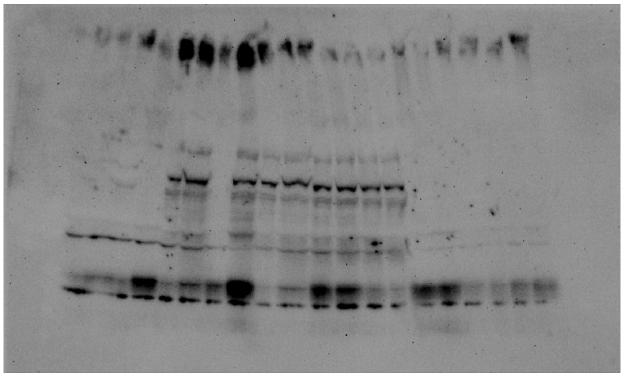

Caspase-1

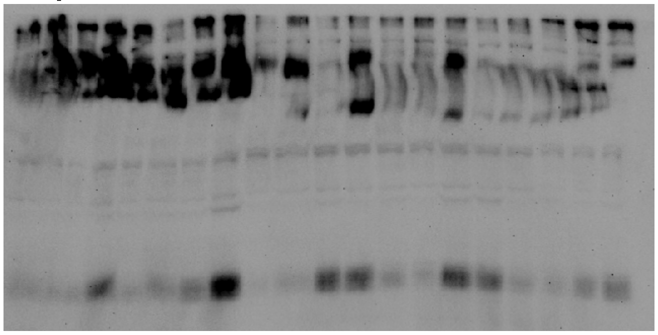

Caspase-11

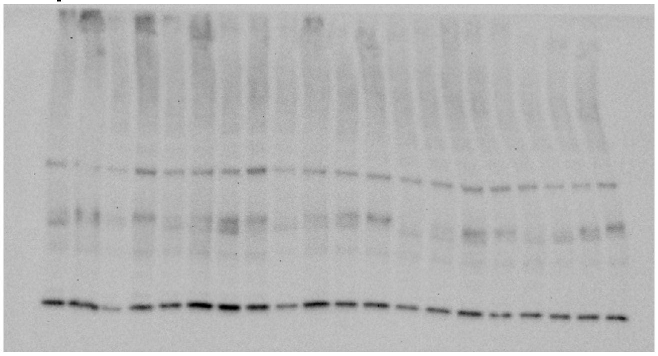

Caspase-8

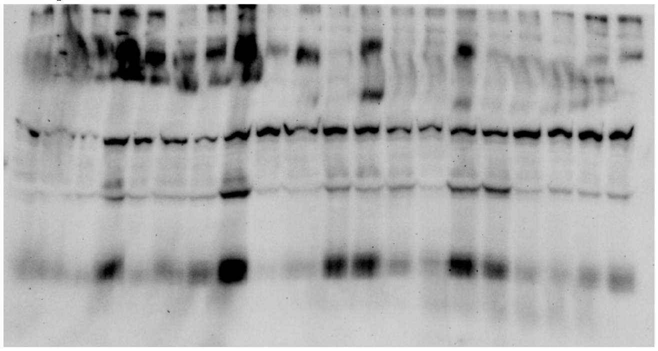

β-Actin

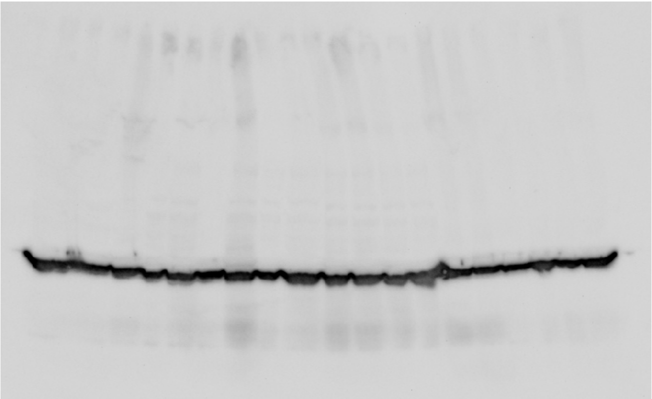

Hippocampus

(WT-SH, WT-TBI, AD-SH, AD-TBI) x 5

ASC

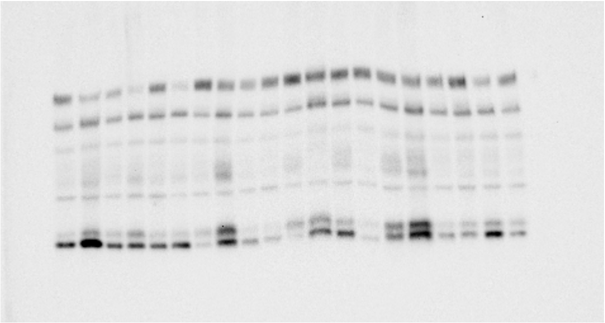

Caspase-8

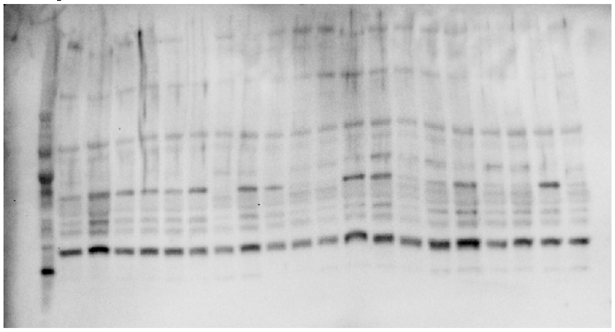

IL-1β

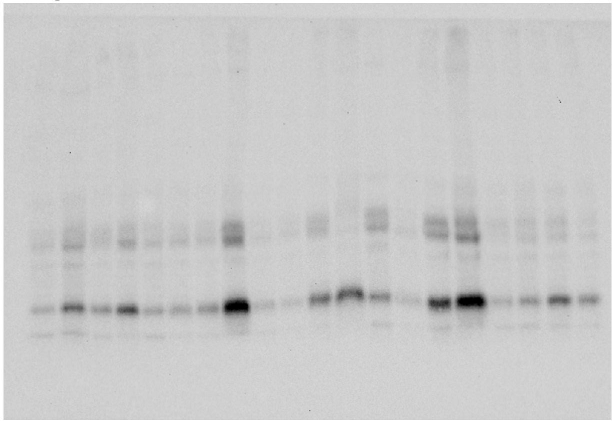

β-Actin

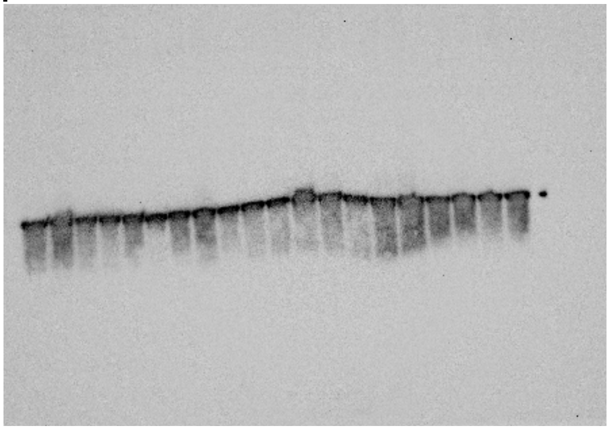

Supplement: Supplementary file 1 [file cells-15-01658-s001.zip › cells-4503640-supplementary.pdf]
